# Variation in the Early Host-Pathogen Interaction of Bovine Macrophages with Divergent Mycobacterium bovis Strains in the United Kingdom

**DOI:** 10.1128/IAI.00385-17

**Published:** 2018-02-20

**Authors:** Kirsty Jensen, Iain J. Gallagher, Nicholas Johnston, Michael Welsh, Robin Skuce, John L. Williams, Elizabeth J. Glass

**Affiliations:** a Division of Infection & Immunity, The Roslin Institute & R(D)SVS, University of Edinburgh, Easter Bush Campus, Midlothian, United Kingdom; b School of Biological Sciences, Medical Biology Centre, Queen's University, Stormont, Belfast, United Kingdom; c Veterinary Sciences Division, AFBI Stormont, Belfast, United Kingdom; d The Davies Research Centre, School of Animal and Veterinary Sciences, University of Adelaide, Roseworthy, Australia; Weill Cornell Medical College

**Keywords:** *Mycobacterium*, cattle, host-pathogen interactions, macrophages

## Abstract

Bovine tuberculosis has been an escalating animal health issue in the United Kingdom since the 1980s, even though control policies have been in place for over 60 years. The importance of the genetics of the etiological agent, Mycobacterium bovis, in the reemergence of the disease has been largely overlooked. We compared the interaction between bovine monocyte-derived macrophages (bMDM) and two M. bovis strains, AF2122/97 and G18, representing distinct genotypes currently circulating in the United Kingdom. These M. bovis strains exhibited differences in survival and growth in bMDM. Although uptake was similar, the number of viable intracellular AF2122/97 organisms increased rapidly, while G18 growth was constrained for the first 24 h. AF2122/97 infection induced a greater transcriptional response by bMDM than G18 infection with respect to the number of differentially expressed genes and the fold changes measured. AF2122/97 infection induced more bMDM cell death, with characteristics of necrosis and apoptosis, more inflammasome activation, and a greater type I interferon response than G18. In conclusion, the two investigated M. bovis strains interact in significantly different ways with the host macrophage. In contrast to the relatively silent infection by G18, AF2122/97 induces greater signaling to attract other immune cells and induces host cell death, which may promote secondary infections of naive macrophages. These differences may affect early events in the host-pathogen interaction, including granuloma development, which could in turn alter the progression of the disease. Therefore, the potential involvement of M. bovis genotypes in the reemergence of bovine tuberculosis in the United Kingdom warrants further investigation.

## INTRODUCTION

Bovine tuberculosis (TB), caused by Mycobacterium bovis, is a disease of cattle of global importance (1). Despite control measures having been in place in the United Kingdom since the 1950s, including a strict test and slaughter policy, the incidence of the disease is increasing in many areas of the country, especially southwest England (2, 3). Additional or alternative methods of control are therefore needed, the development of which will require a greater understanding of the biology of the disease. A number of herd- and animal-level factors have been identified which increase the risk of bovine TB. These include host genetics; *Bos indicus* cattle are more resistant than Bos taurus breeds (reviewed in reference 4). Furthermore, it has been calculated that within the Irish and UK beef and dairy herds, up to 20% of variation in TB resistance is accounted for by cattle genetics (5–9). In addition, regions of the bovine genome have been associated with TB susceptibility in Holstein-Friesian dairy cattle (6, 10–12). Other risk factors include farm management practices, e.g., cattle movement, herd size, environmental factors, and presence of infectious wildlife reservoirs (reviewed in reference 13).

The importance of the genetics of the etiological agent in the resurgence of bovine TB has received relatively less attention, possibly because of the apparently limited variability in M. bovis genotypes. M. bovis is a member of the Mycobacterium tuberculosis complex (MTBC), members of which cause similar pathology in a wide variety of mammals and have evolved from a common ancestor by mutations and deletions with limited evidence of recombination of genetic material in more modern lineages (14). The causative agent of human TB, M. tuberculosis, and the bovine pathogen, M. bovis, are located in different lineages of the MTBC (14), yet they share, on average, 99.95% sequence homology, although the M. bovis genome is smaller due to deletion events (15). The current limited genetic diversity of the M. bovis population in the United Kingdom compared with the diversity of genotypes seen elsewhere, e.g., in France, suggests that it has undergone a population bottleneck, which may be the result of the test-and-slaughter policy restricting variation (16). However, since this bottleneck, M. bovis has diversified in isolation in different geographical regions of the United Kingdom (17), and clonal sublineages can be identified which are the predicted descendants of a common ancestor, identifiable by characteristic mutations and deletions (18). Fortunately, it has been shown that in Northern Ireland the M. bovis genotype does not significantly affect the accuracy of the tuberculin skin test, which is used to detect infected animals (19). However, these genotypes can be separated according to virulence determined by the proportion of skin test-positive animals which have visible granulomas on postmortem inspection (20).

The principal route of M. bovis infection is inhalation into the bovine lung, where the mycobacteria are phagocytosed by alveolar macrophages (Mϕ) (reviewed in reference 21). Within these innate immune cells pathogenic mycobacteria survive and multiply by inducing profound changes in Mϕ biology (reviewed in reference 22). These early interactions are likely to play an important role in determining the progression of the disease. Previous work infecting bovine alveolar Mϕ *in vitro* with virulent and attenuated M. bovis revealed differences in the transcriptional response of infected cells (23). Furthermore, it has been shown that differences in growth of M. bovis isolates in bovine monocyte-derived Mϕ (bMDM) (24) correlated with virulence in a mouse model (25). During infection, differences in the transcriptomic response of these virulent and attenuated M. bovis strains and that of the infected bMDM have been identified (24, 26). Therefore, we hypothesized that M. bovis strains currently circulating in the United Kingdom also exhibit differences in their interaction with host cells, which may influence the pathology of the disease. To further our understanding of the interplay between M. bovis and the host cell, we investigated and compared the infection of bMDM with two UK isolates of M. bovis. The isolates used were the first M. bovis field isolate subjected to bacterial whole-genome sequencing (15), AF2122/97, which is widely considered a reference strain of M. bovis and frequently used in experimental infection studies (27, 28), and strain G18, which is relatively divergent both geographically and genetically from AF2122/97. Significant differences were found in the intracellular growth and survival of the M. bovis strains, the survival of infected bMDM, and the bMDM transcriptional response.

## RESULTS

### Dynamics of M. bovis infection of bMDM.

**FIG 1:**
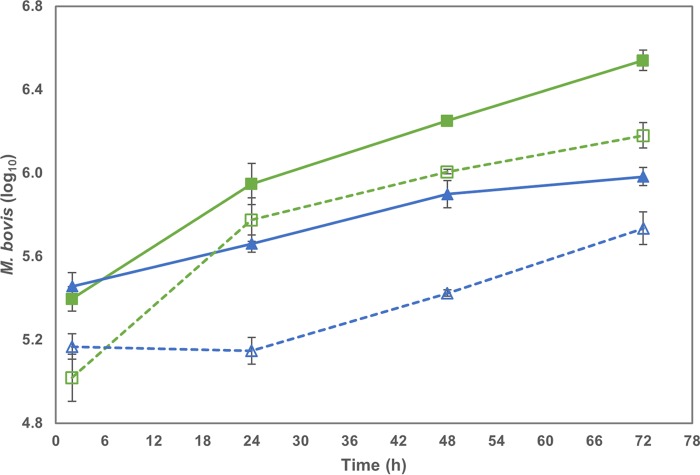
Survival and proliferation of M. bovis strains in bMDM differ. The graph compares the number of M. bovis organisms in bMDM throughout the 72-h time course. M. bovis organisms were quantified by qPCR (solid lines) and CFU (dotted lines) for AF2122 (green squares) and G18 (blue triangles). Error bars denote the standard errors from three biological replicates.

The uptake, survival, and proliferation of the two strains of M. bovis (AF2122/97 and G18) during *in vitro* infection of bMDM were investigated by quantifying the number of intracellular mycobacteria by two methods. Quantification of genome copy number (GCN) by quantitative PCR (qPCR), which does not distinguish between active and inactivated mycobacteria, identified a significant difference in growth rate between these two M. bovis strains (*P* < 0.001) (Fig. 1, solid lines). The numbers of intracellular AF2122/97 (here called AF2122) and G18 were not significantly different 2 h postinfection (hpi), with average M. bovis/bMDM ratios of 0.85 and 0.98, respectively. However, from 24 hpi there were significantly more AF2122 organisms per cell than G18 organisms. The number of AF2122 organisms increased consistently between 24 and 72 hpi, while the increase in G18 numbers lessened between 48 h and 72 h, and at the final time point there were, on average, 3.7 ± 0.5 times more AF2122 organisms per cell than G18 organisms.

Quantification of CFU, which measures the number of active mycobacteria, revealed an overall similar pattern of growth rate differences between the two strains, with no significant difference 2 hpi but significantly different growth rates over the subsequent time course (*P* < 0.001) (Fig. 1, dotted lines). However, the CFU counts were significantly lower than the GCN counts at 2 hpi (*P* < 0.001), with, on average, 0.4 ± 0.1 and 0.5 ± 0.1 CFU per bMDM for AF2122 and G18, respectively. M. bovis bacilli are prone to form clumps, which could lead to an underestimation of the number of CFU and account for this difference. Alternatively, it may relate to the bactericidal activity of bMDM rapidly inactivating the mycobacteria. The quantification by CFU revealed considerable differences in intracellular growth, especially during the first 24 h of infection. AF2122 CFU increased rapidly, so that there were 2.1 ± 0.4 AF2122 organisms per cell by 24 hpi and the GCN/CFU ratio was reduced from 2.9:1 at 2 hpi to 1.5:1 by 24 hpi. AF2122 numbers increased over the remainder of the time course at a similar, but slightly lower, rate than the increase in GCN to give a final GCN/CFU ratio of 2.4:1. In contrast, the average G18 CFU count decreased slightly during the first 24 h, and the GCN/CFU ratio was 3.4:1 at 24 hpi. Thereafter the G18 CFU increased at a higher rate than the GCN, so that the GCN/CFU ratio was reduced to 1.9:1 at 72 hpi. Therefore, the difference in the number of live AF2122 and G18 organisms narrowed throughout the time course, with 4.4 ± 0.7 times more AF2122 than G18 at 24 hpi but only 2.8 ± 0.3 times more at 72 hpi. The CFU quantification revealed that between 2 hpi and 72 hpi AF2122 had undergone approximately four rounds of replication, giving a doubling time of 17.5 h. In contrast, G18 had undergone two rounds of replication in the same period, giving a doubling time of 35 h. However, between 24 hpi and 72 hpi the doubling time of AF2122 was slower than that of G18, at approximately 30 h, with half the replication occurring during the first 24 h of infection. Therefore, the two M. bovis strains vary in their ability to survive and grow in these bMDM, which suggests differences in the host-pathogen interactions occurring during *in vitro* infection. The mechanisms behind these differences may be elucidated by investigating the transcriptional response of bMDM during infection.

### Analysis of RNA-Seq data.

**FIG 2:**
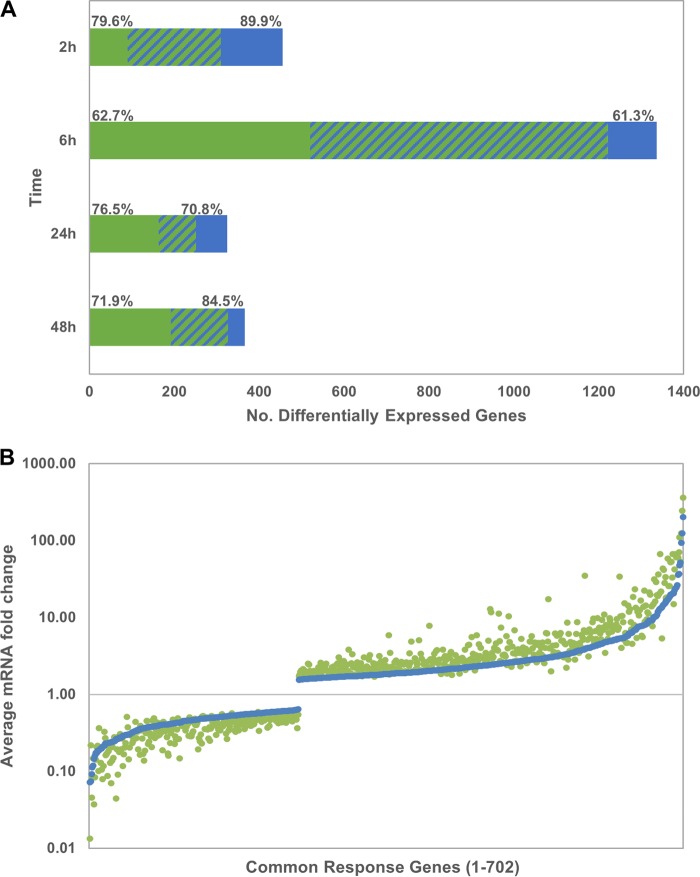
Comparison of the bMDM transcriptional response to M. bovis strains. (A) Graph summarizing the transcriptional response of bMDM to the M. bovis strains at each time point compared to those of the uninfected controls identified by analysis of the RNA-Seq data. The bars represent the number of genes differentially expressed specifically in response to AF2122 (green bars) or G18 (blue bars) and the common transcriptional response (striped bars). The numbers above the green and blue bars denote the percentages of genes that were upregulated in response to AF2122 and G18 infection, respectively, at each time point. (B) Graph illustrating the average fold change quantified by analysis of the RNA-Seq data of 702 differentially expressed genes which overlap the AF2122_6h (green dots) and G18_6h (blue dots) gene lists. The genes are shown in ascending order of differential expression in response to G18.

RNA sequencing (RNA-Seq) was employed to investigate the response of bMDM to infection with the two strains of M. bovis, AF2122 and G18. Differentially expressed genes were identified by comparing their transcriptional levels in response to each M. bovis infection to that in uninfected control samples at each time point (false discovery rate of <1%). The differentially expressed genes are summarized in Fig. 2A and are listed in full in Data Set S1 in the supplemental material. The overall patterns of response to both M. bovis strains were similar. The greatest number of differentially expressed genes was observed 6 hpi. With the exception of 2 hpi, a greater number of genes were differentially expressed in response to AF2122 than G18. In response to both M. bovis strains there was a bias toward upregulated genes at all time points, which was greatest in the G18_2h gene list, with only 10.1% differentially expressed genes being downregulated. The ratios of up- and downregulated genes were most similar in the 6-h gene lists, with 37.3% and 38.7% of differentially expressed genes being downregulated in AF2122_6h and G18_6h gene lists, respectively.

Comparison of the AF2122 and G18 gene lists at each time point revealed that the greatest overlap was in the response of bMDMs to the two M. bovis strains at the earlier time points (Fig. 2A). Approximately half the genes were differentially expressed in response to both AF2122 and G18 at 2 hpi and 6 hpi, with 220 and 702 genes, respectively. The level of overlap in differentially expressed genes dropped at the later time points, 27.2% and 36.9% at 24 hpi and 48 hpi, respectively, indicating that the bMDM responses to the two M. bovis strains diverge with time. However, within the common differentially expressed genes there was further evidence of a greater response to AF2122 than G18. The expression of the common response genes was predominantly affected to a greater extent following infection with AF2122 than G18. This was observed at all four time points, e.g., at 6 hpi the expression of only 86 out of 702 common response genes (12.3%) was altered to a greater degree in response to G18 than AF2122 (Fig. 2B).

Combining the differentially expressed gene lists for infections with both M. bovis strains and comparing the transcriptional response over time suggested that there was a biphasic response of bMDM to M. bovis infection, with an early response (2 h and 6 h) and a later response (24 h and 48 h). The greatest temporal overlap in differentially expressed genes was observed between 24-h and 48-h time points, with 38.9% genes differentially expressed at both time points. In contrast, only 12.0 to 14.4% of genes overlapped between the early and late time points compared with 21.6% between the 2-h and 6-h time points (Fig. S1).

### Functional analysis of the transcriptional response of bMDM to M. bovis infection.

Each list of differentially expressed genes was interrogated using the online Database for Annotation, Visualization, and Integrated Discovery (DAVID) (29, 30) for functional annotation analysis. The ENSEMBL gene identifiers were used to identify overrepresented Gene Ontology (GO) terms and Kyoto Encyclopedia of Genes and Genomes (KEGG) pathways. Overall there was considerable similarity in the overrepresented terms for infection with each M. bovis strain. Table 1 summarizes the overrepresented KEGG pathways at each time point postinfection. Throughout the time course there was a considerable cytokine and chemokine response and evidence of the activation of pathogen-sensing pathways, e.g., Toll-like receptor (TLR), NOD-like receptor, RIG-I-like, and cytosolic DNA sensing pathways. In addition, there was evidence of the activation of intracellular signaling pathways, with the overrepresentation of genes associated with the Jak-STAT and mitogen-activated protein kinase (MAPK) signaling pathways.

**TABLE 1:** KEGG pathways overrepresented in the differentially expressed gene lists highlight similarities and differences in response of bMDM to infection with M. bovis strains^*a*^

Common KEGG pathway	Strain-specific pathway	
	AF2122	G18
2 h		
NOD-like receptor signaling pathway	CAMs	B cell receptor signaling pathway
Cytokine-cytokine receptor interaction	MAPK signaling pathway	
Toll-like receptor signaling pathway		
Chemokine signaling pathway		
RIG-I-like receptor signaling pathway		
Jak-STAT signaling pathway		
T cell receptor signaling pathway		
Cytosolic DNA-sensing pathway		
Apoptosis		
Hematopoietic cell lineage		
Natural killer cell-mediated cytotoxicity		
Adipocytokine signaling pathway		
6 h		
Cytokine-cytokine receptor interaction	p53 signaling pathway	Fc gamma R-mediated phagocytosis
NOD-like receptor signaling pathway		
Chemokine signaling pathway		
Toll-like receptor signaling pathway		
Jak-STAT signaling pathway		
RIG-I-like receptor signaling pathway		
Apoptosis		
B cell receptor signaling pathway		
Adipocytokine signaling pathway		
Cytosolic DNA-sensing pathway		
T cell receptor signaling pathway		
Hematopoietic cell lineage		
Natural killer cell-mediated cytotoxicity		
CAMs		
MAPK signaling pathway		
Fc epsilon RI signaling pathway		
Leukocyte transendothelial migration		
24 h		
Cytokine-cytokine receptor interaction		Complement and coagulation cascades
Chemokine signaling pathway		
Cytosolic DNA-sensing pathway		
NOD-like receptor signaling pathway		
Toll-like receptor signaling pathway		
Hematopoietic cell lineage		
48 h		
Cytokine-cytokine receptor interaction	ECM-receptor interaction	Cytosolic DNA-sensing pathway
NOD-like receptor signaling pathway		Toll-like receptor signaling pathway
Hematopoietic cell lineage		RIG-I-like receptor signaling pathway
Chemokine signaling pathway		Complement and coagulation cascades
CAMs		

a Overrepresented KEGG pathways were identified using the online Database for Annotation, Visualization and Integrated Discovery (DAVID) using a cutoff *P* value of <0.05. CAMs, cell adhesion molecules.

Apoptosis was overrepresented at 2 hpi and 6 hpi in response to both M. bovis strains. However, the p53 signaling pathway, which is also involved in cell death, was only overrepresented in the AF2122_6h gene list. Eleven genes associated with this KEGG pathway were present in this gene list, including caspase 3 (*CASP3*), *Fas*, Mdm2, p53 E3 ubiquitin protein ligase homolog (*MDM2*), *MDM4*, sestrin 1 (*SESN1*), and *SESN3*. However, only 3 of these 11 genes, *CD82*, *CASP8*, and *SESN1*, were present in the G18_6h gene list. This suggests that there are differences in host cell survival associated with each M. bovis strain. Fc gamma R-mediated phagocytosis was only identified as an overrepresented pathway in the G18_6h gene list, with 12 genes representing this pathway in the gene list. However, nine of these genes were present in the AF2122_6h gene list, suggesting that this pathway is activated in response to both infections.

The greatest number of differences in overrepresented pathways was observed at 48 hpi. The extracellular matrix (ECM)-receptor interaction pathway was overrepresented in the AF2122_48h gene list. Nine genes associated with this pathway were present in this list, while only three of these were present in the G18_48h gene list. Complement and coagulation cascades were overrepresented in the gene lists for G18 at 24 hpi and 48 hpi. However, many of the genes associated with this pathway were differentially expressed at the same time points in response to AF2122. Although there was not an M. bovis strain-specific effect, these complement and coagulation cascade-associated genes were only differentially expressed at the later time points and therefore represent a later response.

At 48 hpi the pathogen-sensing pathways cytosolic DNA sensing and RIG-I-like receptor signaling were still overrepresented in the response to G18 but not AF2122 (Table 1). Several of the genes associated with these pathways are type I interferon (IFN) response genes, and a large number of type I IFN response genes were present in the differentially expressed gene lists. Of 280 genes induced by IFN-α stimulation of primary murine bone marrow-derived Mϕ (31), 33.9% were present in the gene lists generated from the RNA-Seq data (Table S1). Furthermore, there was a greater number of type I IFN response genes present in the G18_48h gene list than in AF2122_48h, e.g., myxovirus (influenza virus) resistance 1 (*MX1*), which was upregulated 3.1-fold in response to G18 at 48 hpi but not significantly differentially expressed in response to AF2122 at this time point. Furthermore, other type I IFN response genes present in both the AF2122_48 and G18_48 gene lists were upregulated to a higher degree in response to G18 than AF2122 at this time point, e.g., ISG15 ubiquitin-like modifier (*ISG15*), which was upregulated 6.6- and 2.5-fold in response to G18 and AF2122, respectively. However, the reverse pattern was observed at earlier time points, with a greater number of type I IFN response genes being differentially expressed in response to AF2122 than G18, e.g., *MX1*, which was upregulated 3.3- and 3.8-fold in response to AF2122 at 6 hpi and 24 hpi, respectively, but not significantly differentially expressed in response to G18 at these time points (Data Set S1). In addition, other IFN response genes were present in both AF2122 and G18 gene lists at 6 hpi and 24 hpi but were expressed at higher levels during infection with AF2122 than G18, e.g., radical *S*-adenosyl methionine domain containing 2 (*RSAD2*), which was upregulated 17.4- and 19.3-fold at 6 h and 24 h after AF2122 infection, respectively, but only upregulated 3.0- and 2.3-fold after 6 h and 24 h infection with G18, respectively. In contrast, at 48 hpi *RSAD2* was upregulated 2.9- and 9.2-fold in response to AF2122 and G18, respectively. These results suggest that there is a temporal difference in the type I IFN responses of bMDM to infection with different M. bovis strains.

### Validation of the RNA-Seq data.

The expression of 19 genes was investigated by reverse transcription (RT)-qPCR in a different set of samples derived from bMDM generated from a different cohort of animals to confirm the transcriptional response of bMDM to infection with the M. bovis strains. The time course was extended to 72 h to allow further investigation of the divergence in response at the later time points observed in the analysis of the RNA-Seq data. Four genes were chosen for the RT-qPCR analysis, which represented the p53 pathway, three of which, *CASP3*, *FAS*, and *MDM2*, were differentially expressed in response to AF2122 6 hpi. In addition, the expression of the transcription factor tumor protein 53 (*TP53*), also known as p53, was measured. The timing of the type I IFN response appeared to differ between AF2122 and G18 infection of bMDM. To investigate this response, the expression of six genes, chemokine (C-X-C motif) ligand 10 (*CXCL10*), interferon-induced protein with tetratricopeptide repeats 1 (*IFIT1*), *MX1*, *ISG15*, 2′-5′-oligoadenylate synthetase 1, 40/46kDa (*OAS1Y*), and *RSAD2*, was measured. Furthermore, the expression of the type I IFNs, *IFNB1*, *IFNB3*, *IL-6*, and *IFNA*, was investigated. Many genes induced by type I IFNs are also regulated by IFN-γ, the type II IFN, e.g., *CXCL10* and *ISG15* (31). Therefore, the expression of *IFNG* and one of its target genes, indoleamine 2,3-dioxygenase 1 (*IDO1*), was investigated. Finally, the expression of the cytokines *IL1B*, *IL-10*, and tumor necrosis factor (*TNF*) was quantified.

### p53 pathway.

At the earlier time points only *CASP3* and *FAS* were differentially expressed compared with the uninfected controls, and this was observed in response to G18 and not AF2122 (Table 2), which was in contrast to the RNA-Seq data. At 24 hpi and 48 hpi all four genes, *CASP3*, *FAS*, *MDM2*, and *TP53*, were significantly upregulated in response to infection with both M. bovis strains compared to the uninfected control (Table 2, asterisks). However, at 48 hpi and 72 hpi there were significant differences in the expression of all four genes associated with the M. bovis strain (Table 2, daggers), with greater expression in response to AF2122 infection than G18 infection. Therefore, there was a strain-associated difference in the expression of p53 pathway-associated genes, confirming the significance of this pathway identified in the RNA-Seq data analysis. However, the strain-specific differential expression of *TP53* was only observed 72 hpi, later than the differences observed in *CASP3* and *MDM2* expression. This suggests that the transcription factor TP53 does not regulate the expression of these genes and that other pathways must be involved.

**TABLE 2:** Summary of RT-qPCR results quantifying the expression of genes during M. bovis infection of bMDM^*a*^

Gene	M. bovis strain	Strain effect (*P* value)	Result by time postinfection				
			2 h	6 h	24 h	48 h	72 h
TP53	AF2122	NS	0.9 ± 0.1	0.9 ± 0.1	1.4 ± 0.1*	1.4 ± 0.1*	1.9 ± 0.2*†
	G18		0.9 ± 0.1	0.9 ± 0.1	1.9 ± 0.1*	1.6 ± 0.1*	1.2 ± 0.3
CASP3	AF2122	0.001	0.8 ± 0.1	1.0 ± 0.2	4.2 ± 1.6*	6.1 ± 1.4*†	11.6 ± 2.7*†
	G18		0.7 ± 0.1*	0.9 ± 0.1	3.4 ± 0.8*	3.8 ± 0.6*	3.5 ± 1.0*
FAS	AF2122	NS	0.8 ± 0.1	3.6 ± 1.0	4.5 ± 0.5*	4.8 ± 0.7*	9.6 ± 1.4*†
	G18		0.9 ± 0.1	4.2 ± 0.7*	3.7 ± 0.4*	3.1 ± 0.3*	4.1 ± 1.1
MDM2	AF2122	<0.001	1.1 ± 0.1	1.2 ± 0.1	2.6 ± 0.2*	4.3 ± 0.8*†	5.7 ± 0.8*†
	G18		1.1 ± 0.1	1.1 ± 0.1	2.5 ± 0.1*	3.1 ± 0.5*	3.1 ± 0.5*
IFNB1	AF2122	0.021	1.3 ± 0.7	1.3 ± 0.4	1.4 ± 0.4	3.2 ± 0.9	6.0 ± 1.7†
	G18		0.7 ± 0.2	1.6 ± 0.6	1.3 ± 0.4	1.9 ± 0.8	2.0 ± 0.4
IL-6	AF2122	NS	5.3 ± 1.3*	47.9 ± 19.8*	592.5 ± 202.5*	647.8 ± 262.3*	1,916.7 ± 918.9*
	G18		7.4 ± 2.1*	41.6 ± 17.5*	346.7 ± 109.1*	470.1 ± 208.1*	876.5 ± 339.2*
IFNB3	AF2122	NS	2.8 ± 1.3	2.2 ± 1.2	5.0 ± 3.1	4.8 ± 1.1*	17.2 ± 7.5†
	G18		3.0 ± 1.5	2.2 ± 0.7	2.4 ± 0.6	3.4 ± 1.4	2.9 ± 1.0
IFNA	AF2122	0.036	2.2 ± 1.8	1.0 ± 0.4	1.6 ± 0.3	5.5 ± 2.1†	9.2 ± 3.9†
	G18		1.6 ± 0.9	1.5 ± 0.8	1.6 ± 0.7	1.6 ± 0.6	2.4 ± 1.1
CXCL10	AF2122	NS	6.5 ± 5.6	16.0 ± 11.7	69.8 ± 33.1*	5.5 ± 1.1*	4.2 ± 1.8
	G18		4.9 ± 4.0	9.6 ± 7.0	70.0 ± 48.3*	14.0 ± 6.0	21.1 ± 16.1
IFIT1	AF2122	NS	0.7 ± 0.2	1.5 ± 0.6	5.9 ± 2.2	17.9 ± 9.6	22.9 ± 10.5
	G18		0.8 ± 0.2	1.3 ± 0.5	3.6 ± 0.7	3.6 ± 0.7*	12.8 ± 7.8
ISG15	AF2122	0.031	1.0 ± 0.2	2.1 ± 1.0	31.5 ± 7.5*	47.0 ± 21.3*†	39.5 ± 19.1*
	G18		1.1 ± 0.2	2.0 ± 1.2	23.9 ± 7.6*	6.3 ± 1.7*	21.1 ± 9.7*
MX1	AF2122	NS	0.9 ± 0.3	0.9 ± 0.2	5.1 ± 1.7*	9.2 ± 5.4†	7.6 ± 4.2
	G18		1.0 ± 0.3	1.0 ± 0.4	3.2 ± 0.7	1.5 ± 0.2	4.3 ± 2.9
OAS1Y	AF2122	NS	0.9 ± 0.3	0.9 ± 0.2	4.0 ± 0.7*	5.6 ± 2.3†	5.7 ± 2.4
	G18		1.0 ± 0.2	0.9 ± 0.2	3.0 ± 0.5*	1.4 ± 0.3	2.5 ± 1.0
RSAD2	AF2122	0.008	0.9 ± 0.2	5.3 ± 2.4	88.2 ± 41.5*	332.8 ± 255.8*†	174.6 ± 126.7*
	G18		0.9 ± 0.2	3.9 ± 2.3	31.5 ± 5.8*	16.3 ± 2.9*	85.1 ± 65.9*
IFNG	AF2122	<0.001	3.7 ± 3.0	17.9 ± 12.7	227.8 ± 102.4*†	156.5 ± 56.7*†	119.6 ± 40.4*
	G18		1.8 ± 1.2	11.2 ± 8.7	87.1 ± 52.7*	67.9 ± 26.9*	111.4 ± 41.9*
IDO1	AF2122	0.018	2.5 ± 1.5	5.0 ± 2.9	2,363.4 ± 1,032.4*†	2,272.1 ± 837.0*†	523.9 ± 174.0*
	G18		2.0 ± 1.1	4.1 ± 2.1	1,006.5 ± 523.5*	871.3 ± 462.5*	1,025.2 ± 537.1*
IL1B	AF2122	NS	20.4 ± 7.3*	158.9 ± 53.0*	6,615.2 ± 2,246.9*	4,586.5 ± 1,781.4*†	2,579.3 ± 1,024.0*
	G18		25.0 ± 8.5*	148.0 ± 41.5*	2,856.4 ± 5,86.2*	1,751.9 ± 515.0*	2,188.3 ± 924.1*
IL-10	AF2122	<0.001	5.9 ± 1.3*	8.2 ± 4.4	4.1 ± 1.0*	1.0 ± 0.4†	0.4 ± 0.1*†
	G18		6.5 ± 1.1*	9.3 ± 4.9*	9.1 ± 1.5*	4.6 ± 0.8*	2.0 ± 0.6
TNF	AF2122	NS	38.0 ± 18.1*	122.5 ± 34.2*	286.2 ± 55.0*	101.8 ± 22.7*†	82.6 ± 28.7*
	G18		43.7 ± 15.7*	151.3 ± 32.0*	122.3 ± 23.1*	69.8 ± 16.0*	93.4 ± 26.4*

a The results are expressed as the average fold change in mRNA levels in bMDM during infection with M. bovis strains AF2122 and G18 compared to levels of the uninfected controls ± standard errors. An asterisk indicates when the mean fold change values were significantly different from values for resting cells by *t* test corrected for multiple testing (Benjamini-Hochberg) (*P* < 0.05). The effect of M. bovis strain is shown (repeated-measures analysis by GLM with subsequent Fisher's test). NS denotes that there was no significant strain-specific difference in mRNA levels by GLM. Daggers denote time points where there was a significant strain-specific difference in the average mean fold change identified by the time point and strain interaction (GLM with subsequent Fisher's test).

### Analysis of bMDM survival during M. bovis infection.

**FIG 3:**
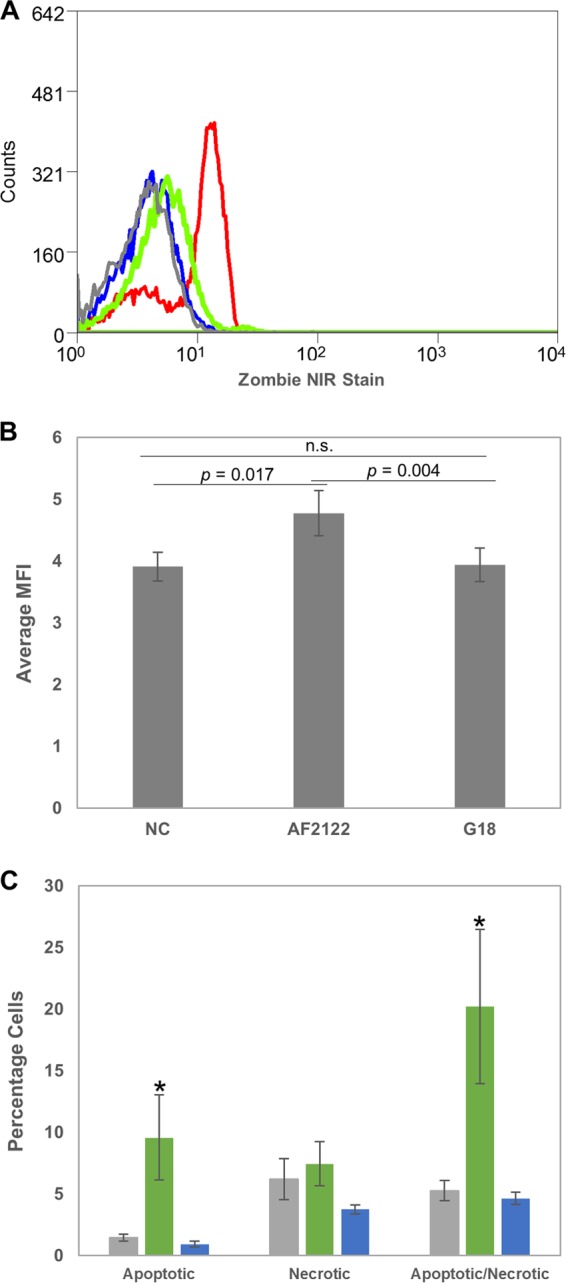
M. bovis strains differ in their effect on bMDM survival. The viability of bMDM was investigated 48 hpi by two methods. First, bMDM were stained with Zombie NIR cell viability dye. (A) Example of a flow cytometry histogram illustrating the shift in staining of bMDM infected with AF2122 (green line) compared to uninfected bMDM (gray line) and those infected with G18 (blue line). The red line illustrates staining of bMDM treated for 30 min with 1% PFA. (B) Histogram illustrating the average mean fluorescence intensities (MFI) of uninfected bMDM (NC) and bMDM infected with M. bovis strains. Error bars denote the standard errors from five biological replicates. The statistical significance of the MFI differences was investigated by *t* test, and the resulting *P* values are indicated in the graph. n.s., not significant. (C) The proportion of bMDM dying by apoptosis and necrosis was investigated. The histogram illustrates the average percentage of bMDM exhibiting markers of apoptosis and necrosis in uninfected cells (gray bars), AF2122-infected cells (green bars), and G18-infected cells (blue bars). Error bars denote the standard errors from five biological replicates. An asterisk denotes that there was a statistically significant difference in the number of bMDM exhibiting that phenotype and that of uninfected bMDM by GLM and subsequent Fisher's test (*P* < 0.05).

AF2122 infection induced higher expression of p53-signaling pathway-associated genes. These genes are also involved in other pathways associated with cell death, e.g., apoptosis. Analysis by light microscopy revealed that bMDM infected with G18 appeared healthier than those infected with AF2122. G18-infected bMDM remained adherent and maintained their morphology, while a greater proportion of those infected with AF2122 detached (data not shown). To investigate if there were differences in cell survival in bMDM infected with the two M. bovis strains, cells were stained with a viability stain 48 hpi, when the altered morphology was observed and the strain-specific differential expression of p53 pathway-associated genes became apparent. bMDM treated with 1% paraformaldehyde (PFA) showed considerable staining with the viability dye (Fig. 3A). However, there was no difference in viability staining of G18-infected bMDM and uninfected bMDM (Fig. 3A and B). In contrast, there was a shift in viability staining in cells infected with AF2122, an example of which is shown in Fig. 3A. Although the viability dye staining was not as strong as that observed with the positive control, the mean fluorescence intensity (MFI) values were significantly higher in cells infected with AF2122 than G18 and uninfected bMDM (Fig. 3B). There was no significant difference in MFI of uninfected and G18-infected bMDM. Zombie stains bind to primary amine groups of proteins and are excluded from live cells. The shift in viability staining suggests that by 48 hpi bMDM infected with AF2122 are dying, with an impaired ability to exclude the viability dye, while those infected with G18 remain healthier, consistent with the data from the transcriptional analysis.

The mechanism of bMDM cell death was investigated by quantifying the number of apoptotic and necrotic bMDM 48 hpi by analyzing the presence of phosphatidylserine on the cell surface and the loss of plasma membrane integrity, respectively. Flow cytometry results for one representative animal are shown in Fig. S2. AF2122 infection did not increase the number of necrotic bMDM but did increase the number of apoptotic bMDM compared with uninfected bMDM and those infected with G18 (Fig. 3C). Furthermore, there were significantly more bMDM simultaneously exhibiting markers of apoptosis and necrosis with AF2122 infection than with either G18 infection or uninfected controls (Fig. 3C). In contrast, G18 infection did not affect the proportion of healthy bMDM, with no significant difference in the number of apoptotic or necrotic cells compared to uninfected bMDM (Fig. 3C), providing further evidence that the G18 M. bovis strain induces less cytotoxicity in bMDM than AF2122.

### IFN response.

The type I IFN response differed across the infection time course in the RNA-Seq data, with a greater response observed to AF2122 infection than G18 infection at earlier time points, which was reversed at 48 hpi. However, significant upregulation of expression of the six type I IFN-regulated genes investigated, *CXCL10*, *IFIT1*, *ISG15*, *MX1*, *OAS1Y*, and *RSAD2*, was only observed from 24 hpi (Table 2) in the repeat experiments. At 24 hpi, when only *RSAD2* was found to be differentially expressed by G18 infection by analysis of the RNA-Seq data (Data Set S1), four genes, *OAS1Y*, *CXCL10*, *ISG15*, and *RSAD2*, were upregulated in response to AF2122 and G18 (Table 2, asterisks), and there was no strain-specific difference in expression in any of the six investigated genes. At 48 hpi, when the RNA-Seq data analysis revealed a greater type I IFN response in bMDM infected with G18 than with AF2122, RT-qPCR analysis revealed strain-specific differential expression of *ISG15*, *MX1*, *OAS1Y*, and *RSAD2*, with significantly greater responses to AF2122 than G18 (Table 2, daggers), e.g., RSAD2 mRNA levels exhibited 332.8- ± 255.8-fold upregulation compared to the uninfected control in response to AF2122 infection but only 16.3- ± 2.9-fold upregulation in response to G18 infection. However, there were no significant differences in expression of the investigated type I IFN response genes at 72 hpi, largely due to the increasing levels of expression in response to G18. Therefore, although there was a shift in the timing of the type I IFN response revealed by the RT-qPCR analysis, there was evidence of a difference in the response to different M. bovis strains, with a greater type I IFN response to AF2122 at 48 hpi and the suggestion of the beginning of a switch at 72 hpi, which may have become more apparent if the time course had been extended further.

The expression of the type I IFN genes was also investigated. None of these genes were identified as being differentially expressed in the RNA-Seq data; however, the duplication of *IFNA* and other type I IFN gene family members in the bovine genome (32) makes alignment of short RNA-Seq reads difficult, which may mean that these genes were missed from the analysis. *IFNB1* and *IFNB3* were only significantly differentially expressed at 72 hpi, with significantly higher expression in response to AF2122 than G18. *IL-6*, also known as *IFNB2*, was upregulated at all time points compared to the uninfected controls, without any strain-specific difference in expression. There are 13 *IFNA* genes, sharing a high degree of sequence homology, located in the bovine genome (32); therefore, generic primers were designed for *IFNA*, which revealed significantly higher expression of *IFNA* in response to AF2122 infection than to G18 infection at 48 hpi and 72 hpi. Therefore, the differential expression of one or more of the IFN-α family members may account for the observed strain-specific differential expression of the investigated type I IFN response genes at 48 hpi.

**FIG 4:**
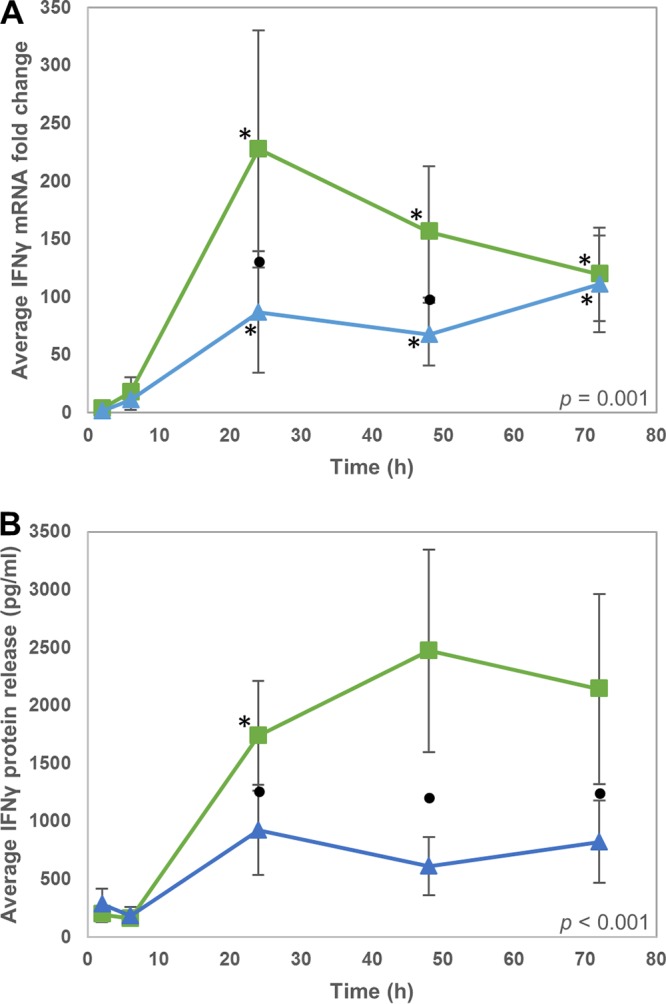
IFN-γ production by bMDM varies in response to infection with different M. bovis strains. (A and B) Average IFN-γ mRNA fold change (A) and IFN-γ protein released (B) from bMDM infected with AF2122 (green squares) and G18 (blue triangles). Error bars denote the standard errors from five biological replicates. An asterisk denotes that the expression at that time point is statistically significantly different from the level of IFN-γ in uninfected bMDM. Black circles denote time points where there is a statistically significant difference in IFN-γ mRNA or protein levels in response to the two different M. bovis strains. The statistical significance of M. bovis strain over the whole time course is indicated on each graph.

The type II IFN gene *IFNG* was also investigated due to overlap in regulation of genes by type I and type II IFNs. In contrast to the type I IFNs, *IFNG* was differentially expressed from 24 hpi in response to infection with both M. bovis strains and exhibited strain-specific differential expression at 24 hpi and 48 hpi, with greater expression in response to AF2122 infection than G18 infection (Table 2 and Fig. 4A). This difference was also seen at the protein level (Fig. 4B), with significantly higher release of IFN-γ from bMDM infected with AF2122 than G18 (*P* < 0.001). Furthermore, expression of *IDO1*, which is stimulated by IFN-γ, also exhibited M. bovis strain-specific differential expression at 24 hpi and 48 hpi (Table 2). Therefore, the strain-specific differential expression of IFN-γ may partly explain the differential expression of some type I IFN response genes which are also regulated by IFN-γ, e.g., *ISG15*.

### Expression of proinflammatory cytokines IL-1β and TNF and the anti-inflammatory cytokine IL-10.

**FIG 5:**
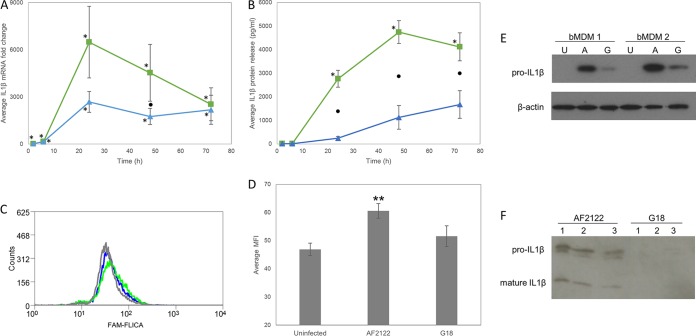
IL-1β production varies in response to different M. bovis strains. (A and B) Average IL-1B mRNA fold change (A) and average IL-1β protein release (B) by bMDM infected with AF2122 (green squares) and G18 (blue triangles). Error bars denote the standard errors from five biological replicates. An asterisk denotes that the expression at that time point is statistically significantly different from the level of that transcript or protein in uninfected bMDM. Black circles denote time points where there is a statistically significant difference in IL-1β mRNA or protein levels in response to the two different M. bovis strains. The statistical significance of M. bovis strain over the whole time course is indicated on each graph. (C) Example of a flow cytometry histogram illustrating the shift in FAM-FLICA staining, indicative of CASP1 activation, of bMDM infected with AF2122 (green line) and G18 (blue line) compared to uninfected bMDM (gray line). (D) Histogram illustrating the average mean fluorescence intensities (MFI) of uninfected bMDM (NC) and bMDM infected with M. bovis strains. Error bars denote the standard errors from four biological replicates. Two asterisks denote that there was a statistically significant difference in CASP1 activity compared to that of uninfected bMDM by GLM and subsequent Fisher's test (*P* < 0.001). (E) Western blot illustrating pro-IL-1β protein levels in bMDM cell lysates 24 h after M. bovis infection. U denotes uninfected, and A and G denote bMDM infected with AF2122 and G18, respectively. bMDM 1 and 2 relate to bMDM isolated from different biological replicates. β-Actin was included as a loading control. (F) Western blot illustrating pro-IL-1β and mature IL-1β protein present in bMDM supernatants collected 48 h after M. bovis infection. Numbers relate to bMDM isolated from different biological replicates.

IL-1β mRNA levels were upregulated throughout the time course in response to infection with both M. bovis strains (Table 2 and Fig. 5A). However, maximal IL-1β mRNA levels were observed at 24 hpi, which was later than that seen in the RNA-Seq data. The measured IL-1β mRNA levels over the whole time course were not significantly different in response to AF2122 or G18 (*P* = 0.071), but there was a statistically significant strain-specific difference in IL-1β expression at 48 hpi. The production and release of mature IL-1β protein were investigated by enzyme-linked immunosorbent assay (ELISA). IL-1β protein was released in response to infection with both M. bovis strains, suggesting that inflammasomes, multiprotein complexes that process the immature pro-IL-1β into the active form, are activated by infection. However, due to the high degree of variability in the amount of IL-1β released by bMDM from different animals in response to G18 infection, the amount produced was not statistically significantly different from that of uninfected cells. Moreover, significantly more IL-1β protein was released in response to AF2122 infection than G18 infection from 24 hpi onwards (*P* < 0.001) (Fig. 5B). At 24 hpi, over 11 times more IL-1β protein was released in response to AF2122 infection than G18 infection (*P* < 0.05) (Fig. 5B). In contrast, the average fold change in IL-1β mRNA level in response to AF2122 infection was 2.1-fold higher than that induced by G18 infection at 24 hpi, which was not a significant difference (Fig. 5A). Therefore, the modest difference in IL-1β mRNA levels observed by 24 hpi cannot account for the great divergence in IL-1β protein being produced in response to different M. bovis strains. The detection of a greater amount of IL-1β protein in the supernatants of AF2122-infected bMDM may relate to the release of pro-IL-1β by the higher proportion of dying cells during AF2122 infection. Alternatively, this disparity in mRNA and protein levels suggests that inflammasomes were activated earlier and to a greater extent in response to AF2122 than G18 infection.

Inflammasome activity during M. bovis infection was investigated by measuring CASP1 activation. Although the shift in fluorescence was relatively minor, probably due to the signal being masked by the highly autofluorescent nature of bMDM, significantly more activated CASP1 was detected in bMDM infected for 24 h with AF2122 than G18 (Fig. 5C and D). However, CASP1 activation during G18 infection was not statistically significantly higher than that observed in uninfected bMDM due to the level of variation in the biological replicates, as with the IL-1β ELISA results. To confirm inflammasome activity in M. bovis-infected cells, IL-1β protein levels in cell lysates and supernatants were investigated by Western blotting. Pro-IL-1β protein levels were consistently greater in AF2122-infected bMDM than in G18-infected bMDM (Fig. 5E), confirming the IL-1β mRNA results (Fig. 5A). Mature IL-1β was not detected in cell lysates; however, both pro-IL-1β and mature IL-1β were detected in cell supernatants (Fig. 5F). The antibodies used in the IL-1β ELISA were found to detect both mature and pro-IL-1β (data not shown), and therefore the presence of both proteins contributes to the ELISA result (Fig. 5B). Pro-IL-1β is presumably released by cells dying in response to AF2122 infection and accounts for the greater proportion of IL-1β present in the supernatants. However, mature IL-1β was consistently detected in supernatants from AF2122-infected bMDM, while little or no mature IL-1β was detected in supernatants from G18-infected bMDM (Fig. 5F), even though pro-IL-1β is produced by these cells (Fig. 5E). These results provide further evidence that M. bovis strains differ in their ability to stimulate host cell inflammasomes.

**FIG 6:**
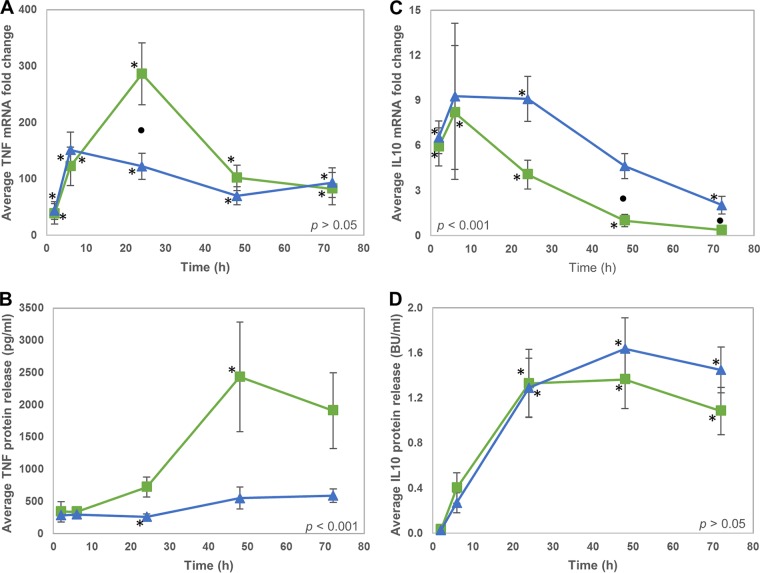
TNF and IL-10 production varies in response to different M. bovis strains. (A to D) Average TNF (A) and IL-10 (C) mRNA fold change and average TNF (B) and IL-10 (D) protein release by bMDM infected with AF2122 (green squares) and G18 (blue triangles). Error bars denote the standard errors from five biological replicates. An asterisk denotes that the expression at that time point is statistically significantly different from the level of that transcript or protein in uninfected bMDM. Black circles denote time points where there is a statistically significant difference in mRNA or protein levels in response to the two different M. bovis strains. The statistical significance of M. bovis strain over the whole time course is indicated on each graph.

As with IL-1β, mRNA levels of the proinflammatory cytokine TNF were significantly upregulated compared to those of the uninfected controls throughout both infection time courses (Table 2 and Fig. 6A). TNF mRNA levels were upregulated to similar extents in response to both M. bovis strains, except at 24 hpi, when there was, on average, over two times higher expression in response to AF2122 than G18. The greater production of TNF in response to AF2122 infection was also observed at the protein level (Fig. 6B), which was statistically significantly different from that produced in response to G18 infection over the 72-h time course (*P* < 0.001).

IL-10 mRNA levels were elevated at 2 hpi and 6 hpi in response to both M. bovis strains in the RNA-Seq experiment. However, in the validation samples, IL-10 mRNA levels were elevated over the whole 72-h infection period, with maximal expression observed at 6 to 24 hpi (Fig. 6C). There was no significant difference in IL-10 mRNA levels induced by AF2122 and G18 infection up to 24 h, but from 6 h onwards IL-10 mRNA levels returned to baseline levels more rapidly following AF2122 infection than G18, resulting in significantly higher levels of IL-10 mRNA at 48 hpi and 72 hpi in bMDM infected with G18. This pattern was also observed at the protein level, although the difference between M. bovis strains did not reach statistical significance (Fig. 6D).

## DISCUSSION

Pathogenic mycobacteria, especially M. tuberculosis and M. bovis, are responsible for arguably the most important bacterial diseases of humans and domestic animals occurring around the globe. These mycobacteria invade alveolar Mϕ, where they adapt the host cell to form a niche where they can survive, protected from the host's immune response. Previously, variation in the response of infected Mϕ to different mycobacteria has been reported, although most of this work has compared virulent and highly attenuated strains of mycobacteria, e.g., the vaccine strain of M. bovis Bacille Calmette-Guerin (BCG) (23, 33). We wished to investigate if early events during Mϕ infection would vary with different M. bovis field strains isolated from infected cattle from the United Kingdom (20, 27). Therefore, we compared various aspects of the early interaction of bMDM with two extant strains of M. bovis, G18 and AF2122, which are genetically relatively divergent among the UK M. bovis population. AF2122 reached higher intracellular numbers during the first 72 h following *in vitro* infection of bMDM than G18 and had more profound effects on the host Mϕ, inducing more cell death and a greater transcriptional response.

The survival and replication of M. bovis during bMDM infection were measured by two methods: qPCR quantification of GCN and conventional CFU counts. The initial uptake of the two M. bovis strains was similar, but during the first 24 h the number of viable G18 organisms was static, while AF2122 numbers increased. However, G18 was replicating during this apparent hiatus, as revealed by the increasing GCN, although at a lower rate than AF2122. Therefore, the dynamics of M. bovis growth and survival were only revealed by combining simultaneous quantification of CFU and GCN, illustrating that G18 was able to replicate but the bMDM were initially killing the mycobacteria at the same rate as G18 replication. However, the ability of bMDM to constrain G18 numbers was lost after 24 h. Overall the results show that AF2122 has the ability to replicate at a higher rate than G18 in these bMDM and is better able to evade Mϕ mycobactericidal activity.

Analysis of the RNA-Seq data suggested that the major transcriptional response of bMDM to M. bovis infection occurred at 6 hpi, within this early phase of infection when the dramatic difference in survival and replication of the two M. bovis strains was observed. Overall the transcriptional responses to infection with the two M. bovis strains were very similar, but there were differences in the intensity of the response with respect to the number of genes and changes in the level of expression measured at this early time point. Therefore, we hypothesized that these differences could account for the variation in AF2122 and G18 growth rates during the first 24 h of infection. However, the subsequent validation work, using bMDM generated in the same way as those used in the intracellular growth assay, revealed a temporal shift in the response of all the investigated genes. M. bovis strain-specific differential expression was first observed 24 hpi, when bMDM had lost that ability to control G18 growth, and the greatest transcriptional response occurred between 24 and 48 hpi, similar to that observed in a recent study investigating the response of bovine alveolar Mϕ to AF2122 infection (34). However, the RNA-Seq and subsequent validation work were in agreement that AF2122 induces a much greater transcriptional response in bMDM than G18. This conclusion is reinforced when it is considered that, although temporally shifted, this greater response was observed in bMDM generated by similar, but distinct, methods from cells isolated from unrelated animals, using different batches of mycobacteria, and using different infection methodologies.

Analysis of the RNA-Seq data identified differential regulation of genes associated with the p53-signaling pathway during infection with the two M. bovis strains. Subsequent analysis confirmed that several genes in this pathway, which are also involved in the regulation of cell death, e.g., *CASP3* and *MDM2*, were induced at significantly higher levels in response to AF2122 infection than G18, which correlated with an increase in the death of AF2122-infected bMDM. Early apoptosis of Mϕ infected with avirulent mycobacteria has previously been reported (35). However, the cell death induced by AF2122 had characteristics of apoptosis and necrosis, with detectable phosphatidylserine on the cell surface and loss of membrane integrity, respectively. This type of cell death has previously been described in association with high intracellular mycobacterial loads (36–39). It is unclear if this is a distinct form of necrosis or if apoptotic cells rapidly progress to secondary necrosis. The fact that AF2122 induces an increase in apoptotic cells as well as bMDM with markers of apoptosis and necrosis suggests that the latter is the case. However, the apoptosis is atypical, being independent of caspases and cathepsins (38). It was originally thought that mycobacteria remained within the modified phagosome in Mϕ, but it is now known that some mycobacteria can escape into the cytoplasm, and this is a major trigger for necrosis of the infected Mϕ (39–41). Apoptosis of infected Mϕ results in mycobacteria being trapped within apoptotic bodies, and when these are engulfed by uninfected Mϕ the mycobacteria are unable to prevent lysosome fusion, as they do during primary infection, which leads to their death (42). In contrast, necrosis promotes bacterial survival and proliferation, allowing the release of mycobacteria into the extracellular environment, which are then able to successfully infect naive Mϕ.

The presence of more mycobacteria, or at least more mycobacterial components, in the cytoplasm of AF2122-infected bMDM is supported by three additional observations. First, a greater type I IFN response was observed during AF2122 infection, with higher expression of IFN-α and IFN-β proteins, along with the downstream type I IFN response genes, e.g., *MX1* and *RSAD2*. Type I IFNs are induced in response to stimulation of pathogen recognition receptors, many of which are cytosolic, e.g., NOD2, cGAS, and RIG-1 (43). The ESX-1 secretion system of M. tuberculosis, which enables effector proteins to be secreted into the host cell cytoplasm, has been shown to be important in the induction of the type I IFN response (44). Second, the initial levels of upregulation of TNF produced in response to AF2122 and G18 infection were similar, but at 24 hpi there was a spike in production in AF2122-infected cells. The activity of the intracellular receptor NOD2 is responsible for most TNF produced by human MDM in response to M. tuberculosis and BCG (45). The presence of AF2122 or AF2122 components in the cytoplasm may explain the elevation in TNF production. Third, there was greater activation of inflammasomes during AF2122 infection than G18 infection, resulting in the production and release of a greater amount of mature, active IL-1β protein. IL-1β protein produced by human MDM in response to M. tuberculosis infection has been shown to be proportional to the infection dose (38). Therefore, the greater mycobacterial burden of AF2122 in bMDM may account for many aspects of the observed differences in the M. bovis-bMDM interaction observed in this study. The AF2122 burden reaches a threshold that induces translocation into the bMDM cytoplasm, which in turn induces bMDM cell death and activation of cytoplasmic receptors, leading to greater inflammasome activation and type I IFN responses. These in turn may act by feedback loops to alter the bMDM transcriptional response.

The mechanisms underlying the ability of AF2122 to replicate at higher rates in bMDM than G18, associated with faster replication and less mycobacterial death, are currently unknown. One mechanism may be that AF2122 regulates the production of nitric oxide by bMDM, which has been shown to limit M. bovis growth in bMDM (46). The G18 genome has not been sequenced to date, unlike that of AF2122 (15). A comparison of the genome sequences and transcriptomes of several M. bovis strains representing the major lineages circulating in Great Britain, including AF2122 and an isolate similar to G18, identified strain-specific differential expression of over 50 M. bovis genes during the first 24 h of alveolar Mϕ infection (47), which may influence early events in the M. bovis-Mϕ interaction. However, the growth of these M. bovis strains in alveolar Mϕ was not compared in this study. Comparison of the AF2122 and G18 genome sequences in conjunction with our knowledge of their growth and survival in bMDM and the host transcriptional response may in the future help to elucidate the mechanisms that promote AF2122 growth while restricting G18.

The comparison of the interaction between bMDM and the two M. bovis strains described in this study revealed that they use different strategies during infection of bMDM. AF2122 employs a hit-and-run approach, with rapid growth associated with a profound effect on the biology of the infected bMDM, while G18 takes a more stealthy approach, with slower mycobacterial growth and lower bMDM responses. Higher levels of the anti-inflammatory cytokine IL-10 induced by G18 than AF2122 may play an important role in the more silent infection of G18, which is currently being investigated (K. Jensen and E. J. Glass, unpublished data). Unfortunately, a direct comparison of the pathologies induced by these M. bovis strains has not been made, and therefore it is not known if these differences in host-pathogen interactions affect the development of disease in cattle. Growth of M. bovis and M. tuberculosis in Mϕ has previously been correlated with pathogenicity in mouse models (25, 48), which would imply that AF2122 is more virulent than G18. This is supported by comparisons of the transcriptional responses of virulent and attenuated M. bovis strains. Infection of alveolar Mϕ by virulent M. bovis induced greater transcription of several chemokines, e.g., chemokine (C-C motif) ligand 5 (*CCL5*) and chemokine (C-X-C motif) ligand 1 (*CXCL1*), than that induced by an attenuated isogenic strain (23). Transcription of several of these chemokines was greater in response to AF2122 than G18 infection. Furthermore, many genes found to be more highly expressed in bMDM infected with virulent M. bovis than with BCG (33) were upregulated to a greater extent during AF2122 infection than G18 infection. Conversely, many of the genes expressed at higher levels in BCG-infected bMDM than those infected with virulent M. bovis were downregulated to a greater degree by AF2122 infection than G18 infection. However, there are insufficient data available at present to assess whether the response of G18 is typical of UK M. bovis strains and AF2122 is hyperstimulatory or if G18 is the outlier.

AF2122 and G18 are both considered virulent, having been isolated from tuberculin skin test reactor cattle in southwest England and Northern Ireland, respectively. Phylogenetic analysis of M. bovis isolates has shown that the same major phylogenetic lineage is present throughout the United Kingdom and Ireland and forms a linear phylogeny with AF2122 at the terminal branch in a sublineage designated B (17). AF2122 contains more unidirectional mutations than other M. bovis sublineages sampled, e.g., sublineage P represented by G18, which implies that the ancestors of AF2122 evolved more recently than those of G18. Challenge studies have shown that AF2122 is highly virulent, and an infection dose of less than 10 bacilli results in pathology in 50% of infected cattle, with the development of large granulomas, aggregates of immune cells surrounding the infected Mϕ which are indicative of mycobacterial infection (27, 28). On average, these granulomas occupy nearly two-thirds of the lymph node where they establish (27). G18 was among the least virulent strains of M. bovis found in Northern Ireland with respect to the proportion of infected animals with visible granulomas (20). This may be related to the more silent infection of Mϕ by G18 seen in this study, involving slower replication of the mycobacteria and more limited signaling by the infected Mϕ to attract other immune cells to form the granuloma. Conversely, the greater transcriptional response induced by AF2122 may attract more immune cells to the site of infection, which, along with greater necrosis and higher mycobacterial numbers, might result in the increased foci of infected cells and the development of larger granulomas.

It is interesting to postulate that the interaction of AF2122 and G18 with bovine Mϕ and subsequent granuloma formation affect the transmissibility of these M. bovis strains. It is possible that the rapid growth and dissemination of AF2122 lead to suboptimal immune responses *in vivo*. AF2122 belongs to the SB0140 spoligotype, which is the dominant genotype circulating in the United Kingdom. In contrast, although still circulating in Northern Ireland, the prevalence of G18 is diminishing over time, suggesting that it is relatively less transmissible than some other M. bovis strains. However, there are currently insufficient data available on variation in relative transmissibility among M. bovis genotypes to shed light on this hypothesis at this time (49).

In conclusion, the two investigated M. bovis strains appear to interact in significantly different ways with the host Mϕ *in vitro*. These differences may affect early events in the development of the immune response and pathogenesis, which could in turn alter the progression of the disease. Therefore, the potential relationship between M. bovis genotype and the effect on pathogenesis and epidemiology warrants further investigation.

## MATERIALS AND METHODS

### Animals.

Peripheral blood mononuclear cells (PBMC) were isolated from female Holstein-Friesian cattle maintained at the Agri-Food & Biosciences Institute (AFBI), Belfast, United Kingdom, and The Roslin Institute, University of Edinburgh, Edinburgh, United Kingdom. The cattle were between 1 and 2 years of age and originated from *M. bovis*- and Johne's disease-free farms. All animals were clinically normal, and all experimental protocols were authorized under the UK Animals (scientific procedures) Act, 1986, and performed to Home Office guidelines. In addition, The Roslin Institute's Animal Welfare and Ethics Committee (AWEC) and the AFBI Veterinary Sciences Division (VSD) Ethical Review Committee ensured compliance with all relevant legislation and promote the adoption and development of the 3Rs (replacement, reduction, and refinement).

### Bacterial strains and culture.

Two genotypes of M. bovis isolated in the United Kingdom, AF2122/97 (spoligotype SB0140, lineage B) (15, 17) and G18 (spoligotype SB0129, lineage P) (17, 20), were cloned to single colonies prior to growth to mid-log phase in liquid Middlebrook 7H9 medium containing Tween 80 and supplemented with 10% albumin-dextrose-catalase (ADC). The mycobacteria then were harvested and prepared as single-cell suspensions by vortexing or using a combination of filtration and sonication before being frozen.

### Macrophage culture and challenge.

bMDM for the RNA-Seq analysis were prepared as follows. Peripheral blood was collected from the jugular vein into sterile bags containing sodium heparin (Fannin), and PBMC were separated by density gradient centrifugation using Ficoll-Paque plus (GE Healthcare Life Sciences). The resulting PBMC were seeded at 3 × 10^6^ cells/ml in 75-cm^2^ flasks and grown in Mϕ medium (RPMI 1640 medium [Invitrogen] supplemented with 15% fetal bovine serum [Lonza Biologicals], 20 mM l-glutamine, nonessential amino acids [Invitrogen], 50 mM HEPES, and antibiotics/antimycotics [Invitrogen]). After 2 to 3 h of culture at 37°C and 5% CO_2_, the nonadherent cells were removed and the adherent cells cultured for 9 days in Mϕ medium. The Mϕ medium was replaced after 24 h and then after every 48 to 72 h. The bMDM were detached using cell dissociation solution (Sigma-Aldrich) and replated on day 9. On approximately day 13, the bMDM were harvested as described above and transferred to 25-cm^2^ flasks, seeded at up to 3 × 10^5^ cells/flask, and cultured for a further 24 h before infection. Flow cytometry, using an anti-bovine CD14 antibody (MCA2678GA; Bio-Rad), confirmed that the Mϕ purity exceeded 95% (data not shown).

The bMDM used in all other experiments were derived from different cattle from those used for the RNA-Seq samples and generated using a protocol similar to that described previously (50). In brief, PBMC were cultured in 75-cm^2^ flasks for 2 h in RPMI 1640 medium without serum at 5 × 10^6^ cells/ml, before the medium was replaced with bMDM medium (RPMI 1640 supplemented with 20% fetal bovine serum [FBS], 4 mM l-glutamine, and 50 μM β-mercaptoethanol) with 100 U/ml penicillin-streptomycin. The bMDM medium was replaced on day 7. On day 11 the adherent cells were rigorously washed with phosphate-buffered saline (PBS) and detached with TrypLE Express (Invitrogen). Flow cytometry, using a mouse anti-human CD14 antibody directly conjugated with Alexa Fluor 647 (MCA1568A647T; Bio-Rad), confirmed that the bMDM purity exceeded 94% (data not shown). Purified bMDM were resuspended at 3 × 10^5^ cells/ml in bMDM medium without penicillin-streptomycin, aliquoted into 12-well plates (1 ml/well), and cultured for 72 h before infection on day 14.

Frozen mycobacterial stocks were thawed and prepared in bMDM medium. In all experiments, bMDM derived from each biological replicate were simultaneously infected with one of the M. bovis strain at a multiplicity of infection (MOI) of 5. Medium only was added to the uninfected controls. Flasks containing bMDM for the RNA-Seq time course experiment were rocked every 15 min for 2 h, and the medium was replaced 6 hpi. Plates containing infected bMDM for all other experiments were centrifuged at 300 × *g* for 5 min, and the medium was replaced 1 hpi.

### RNA-Seq experiment.

bMDM derived from six biological replicates were infected with the two strains of M. bovis or left uninfected, and RNA was isolated at 2, 6, 24, and 48 hpi. The medium was removed and the bMDM were lysed with 2.5 ml TRIzol reagent (Invitrogen) for 20 min to ensure mycobacterial inactivation. RNA was extracted at the ARK-Genomics Facility by following the manufacturer's instructions. The mRNA was purified and fractionated to generate fragments with a median size of 180 to 210 nucleotides. These fragments were reverse transcribed using random primers to generate cDNA libraries. The libraries were sequenced as multiplexes, four tagged samples per flow cell lane, on an Illumina Genome Analyzer IIx platform and sequenced using a paired-end protocol of 54 cycles, resulting in 108 nucleotides being sequenced from each mRNA fragment. Quality control (QC) analysis of the data revealed that over 95% of sequences had good quality scores over the entire 54 nucleotides and, on average, six million reads were generated per sample. Furthermore, on average, 80% of sequences mapped uniquely to the bovine genome.

### RNA-Seq data analysis.

After QC, the data were aligned to the bovine genome (UMD 3.1) from Ensembl release 63 using the TopHat aligner (51), and gene counts were generated for each sample using Cufflinks (52). Differential gene expression between challenged and control cells at each time point was assessed using edgeR (53, 54). Briefly, gene counts were filtered to remove genes with zero counts across all samples and with less than one count/million, in line with guidelines published by the edgeR authors (55). Approximately 47% of bovine genes remained after this filtering step. The data set was then normalized using trimmed mean normalization (54). edgeR then was used to model the data across conditions, accounting for the biological pairing between control and challenged samples from the same animal. A likelihood ratio test was used to assess model fit, and where the inclusion of the challenge effect significantly improved the model fit, the gene was called regulated in order to generate lists of genes that were differentially expressed in response to each mycobacterial infection compared to the uninfected controls. The false discovery rate for differentially regulated genes was set at 1%.

### Quantification of intracellular M. bovis.

Genomic DNA (gDNA) was isolated from bMDM after infection with M. bovis at 2, 6, 24, 48, and 72 hpi using the DNeasy blood and tissue kit (Qiagen) by following the manufacturer's instructions, including the pretreatment of samples with lysozyme to maximize recovery of mycobacterial DNA. The abundance of mycobacterial genome copies in each sample was quantified by qPCR by amplification of the M. bovis 85B antigen gene (MY85B) using the oligonucleotides 5′-CATCAAGGTTCAGTTCCAGAGC-3′ and 5′-TATCCCAGCCGTTGTAGTCG-3′. The levels of each gene were quantified by qPCR using the PerfeCTa SYBR green qPCR supermix (Quanta Biosciences). Reactions were carried out in 10-μl volumes containing 1× master mix, 1 μl forward and reverse primers at predetermined optimal concentrations, and 2.5 μl gDNA sample. Amplification and detection of products were carried out using an Mx3000P PCR machine (Stratagene) with the following cycle profile: 95°C for 3 min, followed by 40 cycles of 95°C for 10 s and 60°C for 30 s. The detection of a single product was verified by dissociation curve analysis. Each PCR experiment was carried out in triplicate, and a log_10_ dilution series of a pGEM-T easy (Promega) plasmid containing MY85B sequence of known size and concentration was included to allow extrapolation of gene copy number (GCN) in each sample.

Intracellular mycobacteria were also quantified as CFU. Cells were lysed 2, 6, 24, 48, and 72 hpi with 0.5 ml PBS plus 0.1% Triton X-100 and incubated at room temperature for 10 min. Aliquots of a 10-fold dilution series were plated onto Middlebrook 7H11 agar plates supplemented with 10% oleic albumin dextrose catalase (OADC) and incubated at 37°C until colonies could be accurately counted.

### Validation of the RNA-Seq results by RT-qPCR.

RT-qPCR was used to validate the RNA-Seq data analysis results. A second set of time course experiments was carried out by infecting bMDM with the two strains of M. bovis, and an additional 72-hpi time point was included. RNA was extracted using the ReliaPrep RNA cell miniprep kit (Promega) with an on-column DNase step as directed by the manufacturers. RNA was quantified using a NanoDrop ND-1000 spectrophotometer (Thermo Scientific). First-strand cDNA was reverse transcribed from 250 ng total RNA using oligo(dT) primer and GoScript reverse transcriptase (Promega) according to the manufacturer's instructions. The resulting cDNA was diluted 1:20 for all genes. Oligonucleotides were designed for each gene using Primer3 (56, 57) and Netprimer (Biosoft International) software (see Table S2 in the supplemental material). The qPCRs were carried out as described above. The relative quantities of mRNA were calculated compared to that detected in the time 0 h uninfected controls using the method described previously (58). The RT-qPCR results for RALBP1-associated Eps domain-containing 1 (*REPS1*) were used to calculate differences in the template RNA levels and thereby standardize the results for the genes of interest. *REPS1* was selected by analysis of the RNA-Seq data as a constitutively and moderately expressed gene in all samples, which was confirmed by RT-qPCR.

### ELISA.

Supernatants were collected 2, 6, 24, 48, and 72 hpi. IL-1β and IFN-γ protein levels in the supernatants were quantified using the bovine IL-1β screening kit (Thermo Scientific Pierce) and bovine IFN-γ-specific ELISA kit (Bio-Rad), respectively, by following the manufacturers' instructions. IL-10 protein was quantified by ELISA using the mouse anti-bovine IL-10 antibodies CC318 and CC320 (Bio-Rad) as coating and detecting antibodies, respectively. Both antibodies were used at 2 μg/ml final concentration. TNF protein was quantified by ELISA using the coating antibody CC327 and the detecting antibody CC328 (Bio-Rad) at 4 μg/ml and 2 μg/ml final concentrations, respectively. The same protocol and buffers were used in the IL-10 and TNF ELISAs as those for the IL-1β ELISA. The concentration of IL-10 and TNF was determined using a standard curve of recombinant ovine IL-10 and bovine TNF (kindly provided by Jayne Hope, The Roslin Institute). All ELISAs were carried out using Nunc-Immuno MaxiSorp 96-well plates, with all samples and standards in triplicate. The plates were read at 450 nm, with the reference values at a wavelength of 550 nm subtracted, using a Synergy HT plate reader (BioTek).

### bMDM cell death assays.

bMDM were prepared and infected with both M. bovis strains as described above at an MOI of 5. Cells were harvested using TrypLE Express (Thermo Fisher) 48 hpi, and cell viability and the mechanism of cell death were investigated. bMDM viability was assessed by staining with a predetermined optimal concentration of Zombie NIR fixable viability stain (BioLegend) (excitation/emission wavelengths [Ex/Em], 719/746 nm) for 30 min. bMDM treated with 1% PFA for 30 min were used as a positive control for cell death. bMDM were washed three times before being fixed with 2% PFA for 30 min. The bMDM then were analyzed using a FACSCalibur (BD Biosciences) flow cytometer. bMDM were initially gated by size and granularity to exclude debris, and the amount of viability stain was measured for a minimum of 10,000 events. The mechanism of cell death was investigated using the apoptosis/necrosis kit (Abcam) by following the manufacturer's instructions. Apoptotic and necrotic cells were identified by staining with Apopxin deep red indicator (red fluorescence; Ex/Em 630/660 nm), which detects phosphatidylserine, and nuclear green DCS1 (green fluorescence; Ex/Em 490/525 nm), which labels the nuclei of membrane-permeable cells, respectively. bMDM treated with 1 μM staurosporine for 18 h or 95% ethanol for 30 min were used as positive controls for apoptosis and necrosis, respectively. bMDM were fixed with 2% PFA for 30 min before analysis using an LSR Fortessa (BD Biosciences) flow cytometer. bMDM were initially gated to exclude debris and doublets, and the amount of red and green fluorescence was measured for a minimum of 10,000 events.

### Inflammasome activation.

bMDM were prepared and infected with both M. bovis strains as described above at an MOI of 5. Cells were harvested 24 hpi using TrypLE Express (Thermo Fisher), and inflammasome activity was investigated by measuring active CASP1 using the FAM FLICA caspase 1 kit (Bio-Rad) by following the manufacturer's instructions. bMDM were fixed with 2% PFA for 30 min before the amount of fluorescent staining was measured by flow cytometry analysis.

### Western blots.

Protein extracts were prepared using M-Per (Thermo Fisher Scientific) with protease inhibitors. Equal volumes of supernatants were concentrated using Ultra-0.5 (10K) centrifugal filter devices (Amicon) by following the manufacturer's instructions. Cell lysates and concentrated supernatants were run on 12% SDS-PAGE gels and blotted onto nitrocellulose. Western blot analyses were performed using primary rabbit antibodies against sheep IL-1β (AHP423; 1:250; Bio-Rad) and human β-actin (ab8227; 1:1,000; Abcam). A goat anti-rabbit IgG antibody conjugated with horseradish peroxidase (HRP) (HAF008; 1:1,000; R&D Systems) was used as a secondary antibody with SuperSignal West Pico chemiluminescent substrate (Thermo Fisher Scientific).

### Statistical analysis.

All statistical analyses, other than the analysis of the RNA-Seq data, were carried out using Minitab, version 17. The data were transformed on the log_10_ scale before statistical analyses to stabilize the variance. Changes in gene expression in infected samples compared to resting cells were analyzed by *t* test corrected for multiple testing (Benjamini-Hochberg). Comparison of the responses of bMDM to the two different M. bovis strains across the time course was analyzed using the general linear model (GLM) to allow repeated-measure analysis, fitting biological replicate as a random effect, with time postinfection and M. bovis strain as fixed effects. Subsequent Fisher's tests were used to identify significant differences between M. bovis strains, as well as the interaction between M. bovis strain and time postinfection.

### Accession number(s).

The RNA-Seq data are publicly available at GEO under the accession number GSE104211.

## Supplementary Material

Supplemental material
