# Supplementary material for: Variation in the Early Host-Pathogen Interaction of Bovine Macrophages with Divergent Mycobacterium bovis Strains in the United Kingdom
Source: Infect Immun. 2018 Feb 20;86(3):e00385-17. doi: 10.1128/IAI.00385-17 (PMC5820943; doi:10.1128/IAI.00385-17)
Supplement: Supplemental material [file supp_86_3_e00385-17__index.html]

Supplemental material 

# Variation in the Early Host-Pathogen Interaction of Bovine Macrophages with Divergent Mycobacterium bovis Strains in the United Kingdom

## Supplemental material

- Supplemental file 1 -

  Table S1. Table listing the differentially expressed genes identified by analysis of the RNA-Seq data which were identified as type I IFN response genes by Liu and coauthors. Table S2. Details of the qRT-PCR primers. Fig. S1. Venn diagrams illustrating the temporal overlap in the transcriptional response of BMDM to infection with *M. bovis*. Fig. S2. Flow cytometry analysis of cell death induced by *M. bovis* infection of BMDM.

  PDF, 555K
- Supplemental file 2 -

  Data Set S1. Genes differentially expressed by BMDM infected with *M. bovis* strains AF2122 and G18.

  XLSX, 204K
