# Supplementary material for: Variation in the Early Host-Pathogen Interaction of Bovine Macrophages with Divergent Mycobacterium bovis Strains in the United Kingdom
Source: Infect Immun. 2018 Feb 20;86(3):e00385-17. doi: 10.1128/IAI.00385-17 (PMC5820943; doi:10.1128/IAI.00385-17)
Supplement: Supplemental material [file IAI.00385-17_zii999092314s1.pdf]

Table S1. Table listing the differentially expressed genes identified by analysis of the RNA-Seq data which were identified as type I IFN response genes by Liu and co-authors (1). The level of differential expression at each time-point in response to each *M. bovis* strain, AF2122 and G18, are listed. ns denotes genes that were not significantly differentially expressed at each time-point.

		**AF2122**				**G18**			
**Gene**	**Gene Name**	**2h**	**6h**	**24h**	**48h**	**2h**	**6h**	**24h**	**48h**
ABTB2	ankyrin repeat and BTB (POZ) domain containing 2	3.82	5.85	ns	ns	2.48	2.33	ns	ns
ADAR	adenosine deaminase, RNA-specific	ns	1.92	ns	ns	ns	ns	ns	ns
APOBEC3A	apolipoprotein B mRNA editing enzyme, catalytic polypeptide-like 3A	4.38	33.79	17.14	ns	ns	5.31	ns	ns
ARID5A	AT rich interactive domain 5A (MRF1-like)	2.13	2.26	ns	ns	ns	ns	ns	ns
ARMCX6	armadillo repeat containing, X-linked 6	ns	-2.47	ns	ns	ns	-1.95	ns	ns
ATP10A	ATPase, class V, type 10A	ns	ns	ns	ns	ns	2.15	ns	ns
BATF2	basic leucine zipper transcription factor, ATF-like 2	ns	2.34	ns	ns	ns	ns	ns	ns
CCL2	chemokine (C-C motif) ligand 2	4.45	3.13	ns	ns	2.38	3.04	ns	ns
CCL4	chemokine (C-C motif) ligand 4	13.48	32.92	3.43	ns	6.79	20.31	2.44	2.25
CCL8	chemokine (C-C motif) ligand 8	13.73	18.33	ns	ns	8.45	16.48	ns	ns
CD274	CD274 molecule	ns	4.67	ns	ns	2.13	2.88	ns	ns
CH25H	cholesterol 25-hydroxylase	ns	ns	ns	ns	2.14	3.04	ns	ns
CXCL10	chemokine (C-X-C motif) ligand 10	23.90	60.30	ns	ns	5.93	24.65	ns	7.50
DAXX	death-domain associated protein	ns	1.82	ns	ns	ns	ns	ns	ns
DDX58	DEAD (Asp-Glu-Ala-Asp) box polypeptide 58	ns	2.24	2.90	ns	ns	ns	ns	2.27
DTX3L	deltex 3-like (Drosophila)	ns	2.23	2.35	ns	ns	ns	ns	1.61
EIF2AK2	eukaryotic translation initiation factor 2-alpha kinase 2	ns	2.43	ns	ns	ns	ns	ns	1.56
EPSTI1	epithelial stromal interaction 1 (breast)	ns	2.77	ns	ns	ns	ns	ns	1.78
F3	coagulation factor III (thromboplastin, tissue factor)	4.44	5.15	ns	ns	3.89	4.02	ns	ns
FAM26F	family with sequence similarity 26, member F	5.03	5.10	3.12	2.62	4.05	3.83	2.08	2.15
FGL2	fibrinogen-like 2	ns	ns	2.31	1.78	ns	ns	ns	ns
GRASP	GRP1 (general receptor for phosphoinositides 1)-associated scaffold protein	3.71	ns	ns	ns	4.10	ns	ns	ns

HERC5	hect domain and RLD 5	ns	4.49	2.66	ns	ns	ns	ns	1.70
IFI35	interferon induced protein 35	ns	2.17	ns	ns	ns	ns	ns	ns
IFI44	interferon-induced protein 44	ns	3.41	3.71	ns	ns	ns	ns	2.05
IFI47	interferon gamma inducible protein 47	ns	7.03	ns	ns	ns	ns	ns	ns
IFIH1	interferon induced with helicase C domain 1	4.73	4.86	3.70	1.93	ns	1.92	ns	2.36
IFIT1	interferon-induced protein with tetratricopeptide repeats 1	2.56	5.54	5.08	1.75	ns	2.28	ns	2.74
IFIT2	interferon-induced protein with tetratricopeptide repeats 2	18.49	11.71	5.65	ns	ns	2.43	ns	3.50
IFIT3	interferon-induced protein with tetratricopeptide repeats 3	5.40	7.84	3.98	ns	ns	2.03	ns	4.38
IL10	interleukin 10	8.16	6.69	ns	ns	4.87	4.32	ns	ns
IL15	interleukin 15	5.20	9.11	ns	ns	4.90	4.94	ns	ns
IL6	interleukin 6 (interferon, beta 2)	10.72	11.86	4.54	5.57	6.94	7.45	3.77	3.25
IRF1	interferon regulatory factor 1	5.45	9.77	2.78	1.84	3.55	4.94	1.65	1.58
IRF8	interferon regulatory factor 8	2.15	-1.95	ns	ns	ns	-3.36	ns	ns
ISG15	ISG15 ubiquitin-like modifier	9.09	10.32	10.92	2.48	2.00	2.58	ns	6.62
ISG20	interferon stimulated exonuclease gene 20kDa	3.50	6.55	4.31	ns	ns	ns	ns	ns
KDR	kinase insert domain receptor (a type III receptor tyrosine kinase)	7.82	7.12	ns	ns	2.70	ns	ns	ns
KMO	kynurenine 3-monooxygenase (kynurenine 3-hydroxylase)	ns	3.81	ns	ns	ns	2.66	ns	ns
LRCH1	leucine-rich repeats and calponin homology (CH) domain containing 1	ns	2.12	ns	ns	ns	ns	ns	ns
MB21D1	Mab-21 domain containing 1	ns	2.03	ns	ns	ns	ns	ns	ns
MB21D2	Mab-21 domain containing 2	ns	3.40	ns	ns	ns	ns	ns	ns
MMP13	matrix metallopeptidase 13 (collagenase 3)	ns	ns	ns	ns	ns	ns	ns	39.93
MOV10	Mov10, Moloney leukemia virus 10, homolog (mouse)	ns	2.50	2.34	ns	ns	1.60	ns	ns
MX1	myxovirus (influenza virus) resistance 1, interferon-inducible protein p78 (mouse)	ns	3.30	3.77	ns	ns	ns	ns	3.13
MX2	myxovirus (influenza virus) resistance 2 (mouse)	ns	2.89	5.06	2.05	ns	ns	ns	3.38
MXD1	MAX dimerization protein 1	ns	2.01	2.52	ns	ns	1.90	ns	ns
NAMPT	nicotinamide phosphoribosyltransferase	ns	3.46	ns	ns	ns	1.85	ns	ns
NCOA7	nuclear receptor coactivator 7	ns	ns	ns	ns	1.78	ns	ns	ns
NFIL3	nuclear factor, interleukin 3 regulated	2.21	1.72	ns	ns	ns	ns	ns	ns

NLRC5	NLR family, CARD domain containing 5	ns	2.40	ns	ns	ns	1.73	ns	ns
NMI	N-myc (and STAT) interactor	ns	3.38	ns	ns	ns	2.30	ns	ns
NR4A2	nuclear receptor subfamily 4, group A, member 2	ns	ns	3.63	ns	ns	ns	3.16	ns
OAS1X	2',5'-oligoadenylate synthetase 1, 40/46kDa	ns	2.77	3.51	1.60	ns	ns	ns	2.39
OAS1Y	2',5'-oligoadenylate synthetase 1, 40/46kDa	ns	2.18	2.99	1.61	ns	ns	ns	2.42
OAS2	2'-5'-oligoadenylate synthetase 2, 69/71kDa	ns	2.39	3.11	ns	ns	ns	ns	2.57
P2RY13	purinergic receptor P2Y, G-protein coupled, 13	5.42	ns	ns	ns	5.36	2.96	ns	ns
PARP11	poly (ADP-ribose) polymerase family, member 11	ns	1.79	ns	ns	ns	ns	ns	ns
PARP12	poly (ADP-ribose) polymerase family, member 12	ns	2.23	ns	ns	ns	ns	ns	ns
PARP14	poly (ADP-ribose) polymerase family, member 14	ns	3.16	ns	ns	ns	1.81	ns	ns
PARP9	poly (ADP-ribose) polymerase family, member 9	ns	ns	2.20	ns	ns	ns	ns	1.66
PCGF5	polycomb group ring finger 5	ns	2.03	ns	ns	ns	ns	ns	ns
PELI1	pellino homolog 1 (Drosophila)	ns	2.00	ns	ns	1.68	1.64	ns	ns
PEX26	peroxisomal biogenesis factor 26	ns	ns	ns	ns	ns	-1.93	ns	ns
PIK3IP1	phosphoinositide-3-kinase interacting protein 1	-3.37	-7.73	ns	ns	-3.38	-5.05	ns	ns
PML	promyelocytic leukemia	ns	2.57	2.96	ns	ns	1.63	ns	1.74
PNPT1	polyribonucleotide nucleotidyltransferase 1	ns	2.47	ns	ns	ns	ns	ns	ns
PSMB9	proteasome (prosome, macropain) subunit, beta type, 9 (large multifunctional peptidase 2)	ns	2.35	ns	1.75	ns	1.71	ns	ns
RBM43	RNA binding motif protein 43	2.41	2.86	ns	ns	2.04	2.18	ns	ns
RHOH	ras homolog gene family, member H	10.55	10.92	6.97	2.15	5.06	4.71	ns	ns
RNF213	ring finger protein 213	ns	2.65	2.76	ns	ns	ns	ns	1.93
RSAD2	radical S-adenosyl methionine domain containing 2	8.11	17.36	19.28	2.87	ns	3.01	2.29	9.24
RTP4	receptor (chemosensory) transporter protein 4	ns	2.50	4.36	1.69	ns	ns	ns	2.28
SAMHD1	SAM domain and HD domain 1	ns	-2.04	ns	ns	ns	-1.69	ns	ns
SETDB2	SET domain, bifurcated 2	-2.64	ns	ns	ns	ns	ns	ns	ns
SLAMF8	SLAM family member 8	ns	3.29	ns	2.68	2.05	2.91	ns	ns
SOCS1	suppressor of cytokine signaling 1	4.14	4.58	ns	ns	3.48	2.48	ns	ns
SOCS2	suppressor of cytokine signaling 2	ns	ns	ns	2.38	ns	ns	ns	ns

SP100	nuclear antigen Sp100	ns	2.57	ns	ns	ns	ns	ns	ns
SPRY2	sprouty homolog 2 (Drosophila)	-2.39	ns	ns	-1.77	-1.96	ns	-1.78	ns
SPSB1	splA/ryanodine receptor domain and SOCS box containing 1	ns	1.85	ns	ns	1.67	ns	ns	ns
STAT1	signal transducer and activator of transcription 1, 91kDa	ns	2.02	ns	ns	ns	ns	ns	ns
TAP1	transporter 1, ATP-binding cassette, sub-family B (MDR/TAP)	ns	3.38	ns	1.61	ns	2.05	ns	1.58
TBC1D1	TBC1 (tre-2/USP6, BUB2, cdc16) domain family, member 1	ns	1.86	ns	ns	ns	1.68	ns	ns
TMEM173	transmembrane protein 173	ns	-3.44	ns	ns	ns	-2.19	ns	ns
TMEM2	transmembrane protein 2	ns	2.48	ns	ns	ns	1.80	ns	ns
TNFSF10	tumor necrosis factor (ligand) superfamily, member 10	ns	3.30	4.38	ns	ns	ns	ns	ns
TOR1AIP2	torsin A interacting protein 2	ns	1.93	ns	ns	ns	1.68	ns	ns
TRIM21	tripartite motif containing 21	ns	2.32	ns	ns	ns	ns	ns	ns
TRIM25	tripartite motif containing 25	ns	2.66	ns	ns	ns	ns	ns	ns
UBE2L6	ubiquitin-conjugating enzyme E2L 6	ns	2.19	ns	ns	ns	ns	ns	ns
USP12	ubiquitin specific peptidase 12	ns	2.21	ns	ns	ns	1.82	ns	ns
USP18	ubiquitin specific peptidase 18	ns	4.03	3.69	1.69	ns	ns	ns	2.81
ZBP1	Z-DNA binding protein 1	ns	2.85	4.54	2.03	ns	ns	ns	2.80
ZNFX1	zinc finger, NFX1-type containing 1	ns	2.57	2.86	ns	ns	ns	ns	1.79

Table S2. Details of the qRT-PCR primers.

Gene	Accession No.	Orientation	Primer sequence (5'-3')
2',5'-oligoadenylate synthetase 1, 40/46kDa (OAS1Y)	NM_001040606	F<br>R	AGTTCTCCCCCTGCTTCAC<br>GCTGCTCCTTACACAGTTGG
caspase 3, apoptosis-related cysteine peptidase (CASP3)	NM_001077840	F<br>R	TGATAAGAGCGTGAACTGAAGA<br>TGCCCAACTGACTGACTGA
chemokine (C-X-C motif) ligand 10 (CXCL10)	NM_001046551	F<br>R	AAGGGAAAGGGTGGCTCAT<br>AAGGCTGGGACTTAGCACATT
Fas cell surface death receptor (FAS)	NM_174662	F<br>R	AAGAACGGTATGGAGGAAGC<br>ATTGCGGAGCAGTTGGAC
indoleamine 2,3-dioxygenase 1 (IDO1)	NM_001101866	F<br>R	TGGGCATTCAGCACAGTATT<br>GACCGAGGGCTTTGACTTTA
interferon-induced protein with tetratricopeptide repeats 1 (IFIT1)	XM_015469501	F<br>R	GCTGCCAAGTTTTACCGAAG<br>CAAAGCCCTGTCTGGTGATG
IFN-alpha (IFNA) - generic	NM_001172040-2	F<br>R	AAGCCATCTCTGTGCTCCAC<br>CCCCTCCTCCTGCCTCAG
interferon, beta 1 (IFNB1)	NM_174350	F<br>R	TGAGGAGATGAAGCAAGAACAG<br>GGTGAGAATGCCGAAGATGT
interferon, beta 3 (IFNB3)	NM_001114297	F<br>R	AGCCCTGTGCCTGTTTTCATCA<br>CCGCCTTTGCTGGAATCTGAG
interferon gamma (IFNG)	NM_174086	F<br>R	GCAAGTCTATGGGATTTCAAGG<br>GGCATCATTTCATTTATCAGCA
interleukin 1, beta (IL1B)	NM_174093	F<br>R	TCCGACGAGTTTCTGTGTGA<br>TGTGAGAGGAGGTGGAGAGC
interleukin 6 (IL6)	NM_173923	F<br>R	ACCACTCCAGCCACAAACAC<br>ATGCCCAGGAACTACCACAA
interleukin 10 (IL10)	NM_174088	F<br>R	TGGATGACTTTAAGGGTTAC<br>AGGGCAGAAAGCGATGAC
ISG15 ubiquitin-like modifier (ISG15)	NM_174366	F<br>R	GATCAATGTGCCTGCTTTCC<br>TCAGCCACAGTCTGCTTCAG
MDM2 proto-oncogene, E3 ubiquitin protein ligase (MDM2)	NM_001099107	F<br>R	TCTGGATGCTGGTGTAAGTG<br>TTCTTGTCCTTCTTCGCTGA
myxovirus (influenza virus) resistance 1, interferon-inducible protein (MX1)	NM_173940	F<br>R	CAGGTGGAAAAGGAAATCAG<br>CAGGAAGGTCTATCAGGGTCA
radical S-adenosyl methionine domain containing 2 (RSAD2)	NM_001045941	F<br>R	GGCTTCTGCTTCCACACA<br>TTCTCCATACCTGCTTCTTTCA
tumour necrosis factor (TNF)	NM_173966	F<br>R	GGGACACCCAGAATGTGAG<br>ATTGGCAGGAAGGGAGAGTT
tumor protein p53 (TP53)	NM_174201	F<br>R	GCACCACCATCCACTACAAT<br>CACAAACACGCACCTCAAA
RALBP1 associated Eps domain containing 1 (REPS1)	NM_001193011	F<br>R	AAGCCGAGAAACATCCAGAG<br>ACATTGGCGGGAGCACTA

F and R denote forward and reverse primers respectively

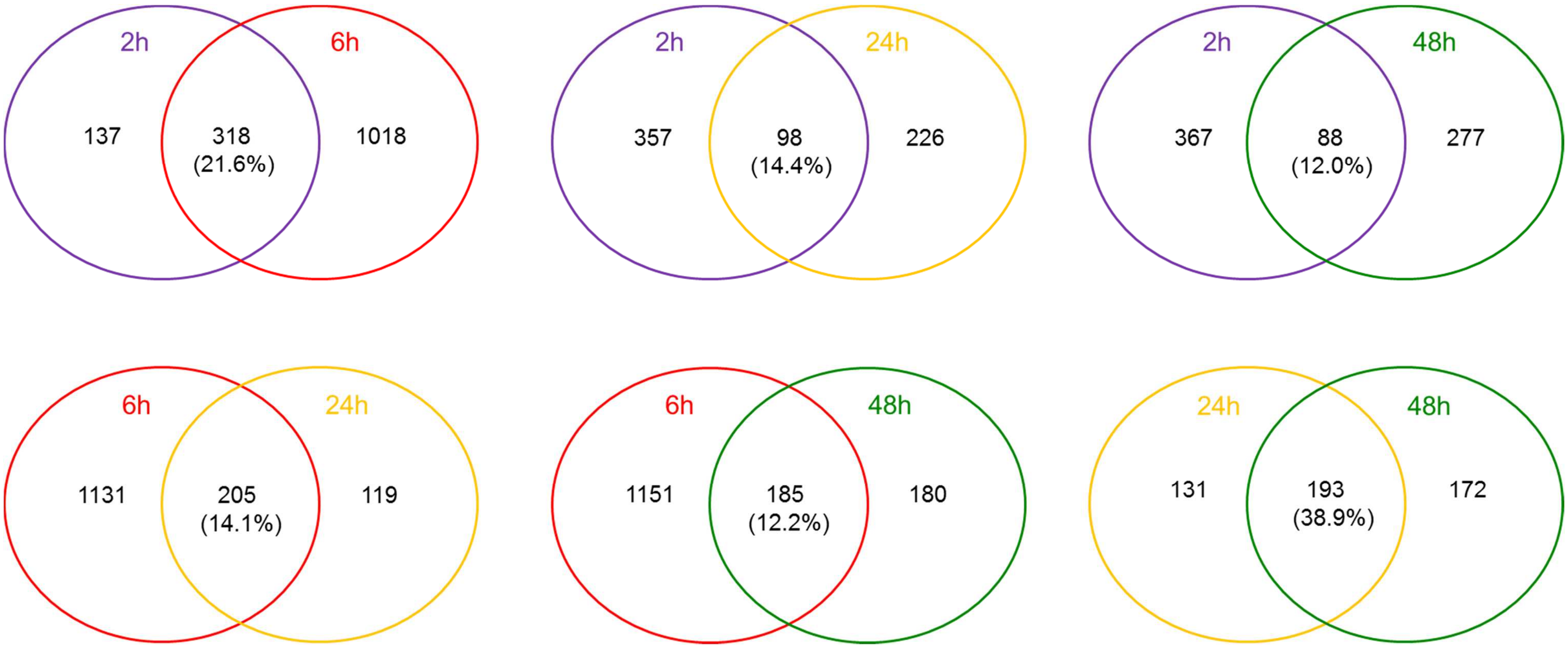

Figure S1. Venn diagrams illustrating the temporal overlap in the transcriptional response of bMDM to infection with *M. bovis*. For each time-point the lists of genes differentially expressed in response to AF2122 and G18 compared to uninfected controls were combined. Venn diagrams were then generated to compare each time-point gene list.

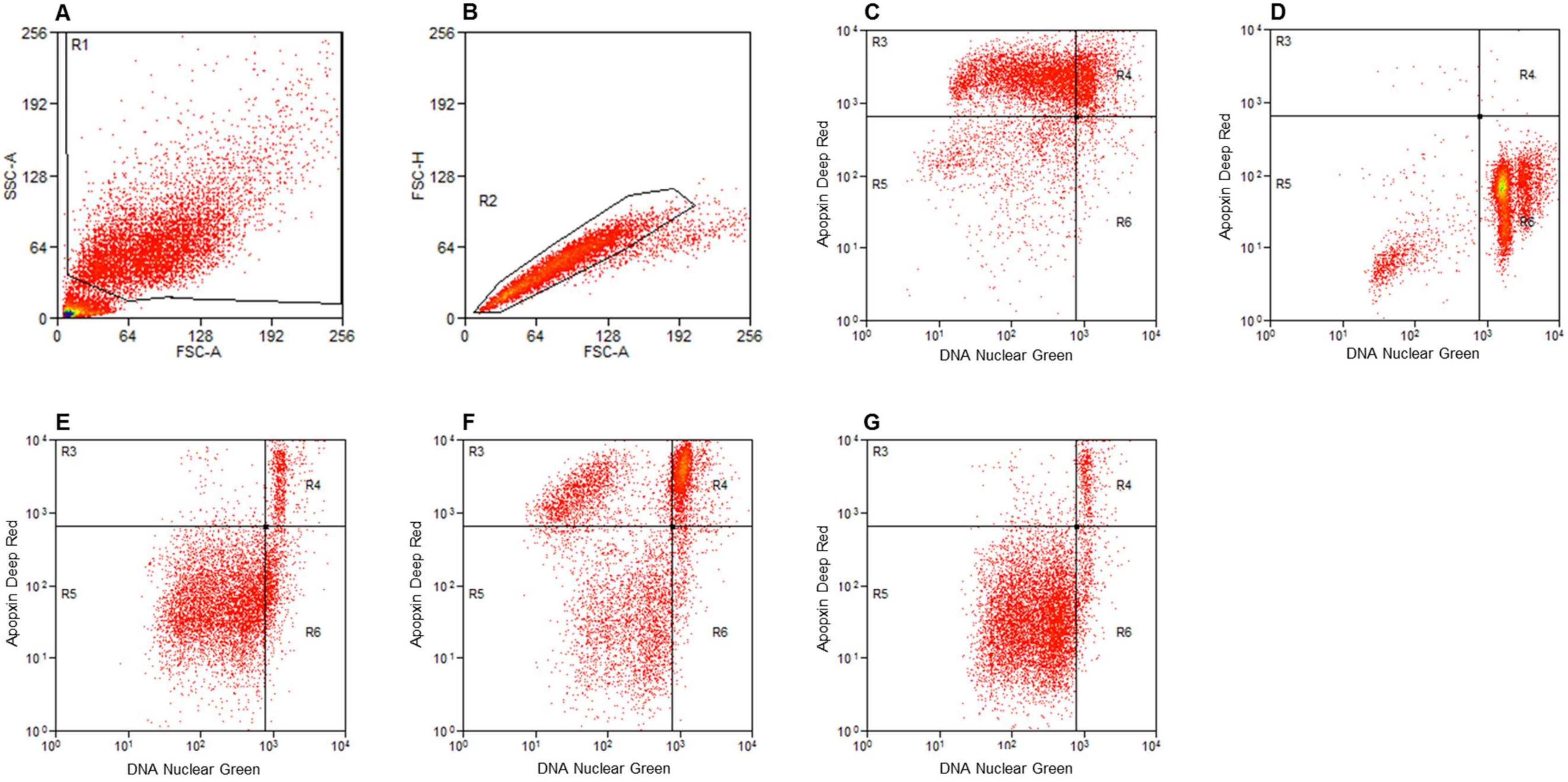

Figure S2. Flow cytometry analysis of cell death induced by *M. bovis* infection of bMDM. bMDM were stained with DNA Nuclear Green, which labels the nuclei of membrane permeable cells, and Apopxin Deep Red, which labels phosphatidylserine, to identify necrotic and apoptotic cells, respectively. The bMDM were initially gated to eliminate debris (A) and doublets (B). Cells were treated with staurosporine (C) or 95% ethanol (D) to induce apoptosis and necrosis, respectively. bMDM were left uninfected (E) or infected with *M. bovis* strains AF2122 (F) or G18 (G) for 48h. The data shown are for one representative animal.

References

1. Liu SY, Sanchez DJ, Aliyari R, Lu S, Cheng G. 2012. Systematic identification of type I and type II interferon-induced antiviral factors. Proc Natl Acad Sci USA 109:4239-4244.
